# A thermodynamically consistent monte carlo cross-bridge model with a trapping mechanism reveals the role of stretch activation in heart pumping

**DOI:** 10.3389/fphys.2022.855303

**Published:** 2022-09-08

**Authors:** Kazunori Yoneda, Ryo Kanada, Jun-ichi Okada, Masahiro Watanabe, Seiryo Sugiura, Toshiaki Hisada, Takumi Washio

**Affiliations:** ^1^ Section Solutions Division, Healthcare Solutions Development Unit, Fujitsu Japan Limited, Shiodome City Center, Tokyo, Japan; ^2^ RIKEN Center for Computational Science HPC- and AI-driven Drug Development Platform Division, AI-driven Drug Discovery Collaborative Unit, Kobe, Japan; ^3^ UT-Heart Inc., Kashiwanoha Campus Satellite, Kashiwa, Japan; ^4^ Graduate School of Frontier Sciences, University of Tokyo, Kashiwanoha Campus Satellite, Kashiwa, Japan

**Keywords:** stretch activation, Monte Carlo method, finite element method, heartbeat, excitation contraction coupling, spontaneous oscillation, cross-bridge cycle

## Abstract

Changes in intracellular calcium concentrations regulate heart beats. However, the decline in the left ventricular pressure during early diastole is much sharper than that of the Ca^2+^ transient, resulting in a rapid supply of blood to the left ventricle during the diastole. At the tissue level, cardiac muscles have a distinct characteristic, known as stretch activation, similar to the function of insect flight muscles. Stretch activation, which is a delayed increase in force following a rapid muscle length increase, has been thought to be related to autonomous control in these muscles. In this numerical simulation study, we introduced a molecular mechanism of stretch activation and investigated the role of this mechanism in the pumping function of the heart, using the previously developed coupling multiple-step active stiffness integration scheme for a Monte Carlo (MC) cross-bridge model and a bi-ventricular finite element model. In the MC cross-bridge model, we introduced a mechanism for trapping the myosin molecule in its post-power stroke state. We then determined the rate constants of transitions for trapping and escaping in a thermodynamically consistent manner. Based on our numerical analysis, we draw the following conclusions regarding the stretch activation mechanism: (i) the delayed force becomes larger than the original isometric force because the population of trapped myosin molecules and their average force increase after stretching; (ii) the delayed force has a duration of more than a few seconds owing to a fairly small rate constant of escape from the trapped state. For the role of stretch activation in heart pumping, we draw the following conclusions: (iii) for the regions in which the contraction force decreases earlier than the neighboring region in the end-systole phase, the trapped myosin molecules prevent further lengthening of the myocytes, which then prevents further shortening of neighboring myocytes; (iv) as a result, the contraction forces are sustained longer, resulting in a larger blood ejection, and their degeneration is synchronized.

## Introduction

**FIGURE 1:**
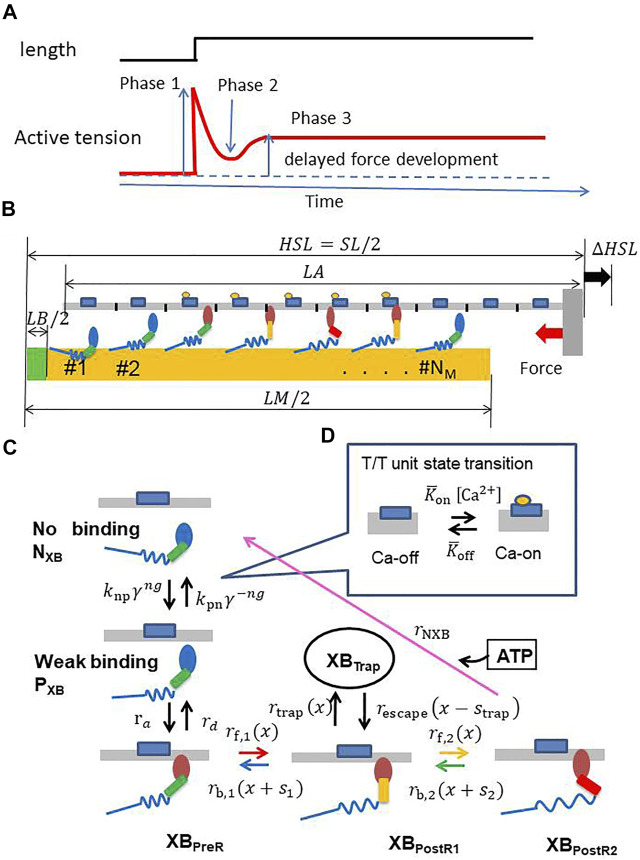
**(A)** A typical stretch activation response for a cardiomyocyte. Time course of the active tension (bottom) is illustrated for the response of length change (1–2%) (top) after steady-state is achieved. There is an initial increase in the active tension with the stretch (Phase 1), followed by a rapid decay in tension (Phase 2) to a minimum, and finally a delayed increase in tension (Phase 3, stretch activation). **(B)** A filament pair in the half-sarcomere model **(C)** the state transition MC model of the myosin molecule, and **(D)** the T/T unit. $N_{\text{M}}$ MHs and $N_{\text{T}}$ T/T units are arranged on the thick and thin filaments, respectively. The MHs in either the N_XB_ or P_XB_ states are assumed to be detached. The rate constant factors $k_{\text{np}}$ and $k_{\text{pn}}$ between N_XB_ and P_XB_ are affected by the state of the T/T unit above it. ng is an integer that takes a value of 0, 1, or 2, according to the number of neighboring MHs attached or weakly binding. γ=80  was adopted to model the cooperativity of the MHs. The forward transitions from XB_PreR_ to XB_PostR2_ *via* XB_PostR1_ are called “power strokes,” whereas the back transitions are called “reverse strokes.” It should be noted that a new state, “XB_Trap_,” is introduced and interacts only with XB_PostR1_. The transition from XB_PostR1_ to XB_Trap_ is called “trapping,” whereas the opposite transition is called “escaping.” The MHs connected to the extremely strained myosin rods are detached (arrows of these forced detachments are not shown).

Stretch activation is a distinctive feature in the tension response that occurs after a small rapid stretch (lengthening of approximately 1% of the initial length) is imposed in the fiber direction to the isometrically contracting muscle. As illustrated in Figure 1A, after the rapid rise of tension during stretching (phase 1), the tension declines to a certain level (phase 2) and then rises again to a level higher than that of the original isometric force (phase 3). In the following, we refer to the force in phase 3 as the delayed force following Stelzer et al. (2006). The degree of delayed force development, or stretch activation, varies for different types of muscles. In particular, an increased delayed force is prominent in asynchronous insect flight muscles, in which stretch activation is thought to be a key factor of the spontaneous oscillations (SPOCs) that occur without intracellular Ca^2+^ regulation (Pringle 1978). Although heartbeats are regulated by the intracellular Ca^2+^ transient ([Ca^2+^]), the stretch activation mechanism is thought to promote efficient switching between the systole and diastole. In our previous work on a numerical bi-ventricular model (Washio et al., 2018), we showed that stretch activation might aid in synchronizing the generation of contraction force over all of the ventricles for a non-uniform rapid rise of [Ca^2+^] during isovolumetric contraction. The stretch activation may also aid in synchronizing the degeneration of contraction force against a non-uniform slow decline of [Ca^2+^] during early diastole. In the simulation, although the total length change of cardiomyocytes in the systole and diastole was approximately 15–20% larger than the length change in the stretch activation, it was shown that the activated force can be generated locally by instantaneous small stretches at the location where the active tension is smaller than the surrounding part because of inhomogeneities of the activation level (Washio et al., 2018). In this simulation study, we assumed a trapping mechanism for strongly binding myosin molecules and modeled this mechanism using a Langevin dynamics model of the power stroke transition. However, the fairly fine time step (∼0.25 ns) used in solving the Langevin equations presented an obstacle in extending this approach for clinical applications. Furthermore, we showed that a multiple-step active stiffness integration scheme that couples the Monte Carlo (MC) model with a larger time step (∼5 μs ) and the continuum had a much higher computational efficiency (Yoneda et al., 2021). Therefore, in this study, we introduce a stretch activation mechanism in the MC model, targeting clinical uses of beating heart simulations.

We briefly present an overview of our sarcomere model (Figure 1B) with the MC cross-bridge model used in this study (Figures 1C,D). We provide details in the Materials and Methods section. We assume that *N*_*M*_ (= 38) myosin molecules are arranged on the thick filament at regular intervals, except for the bare zone, whereas the thin filament is divided into *N*_*T*_ (= 32) segments, termed troponin/tropomyosin (T/T) units. These numbers of myosin molecules and T/T units were determined by assuming that our one-dimensional model corresponds to one of the double spirals of the actual thin filament and surrounding accessible myosin molecules (Washio et al., 2016). A myosin molecule in our cross-bridge model has one non-binding state (N_XB_), one weakly binding state (P_XB_), and four strong binding states (XB_PreR_, XB_PostR1_, XB_PostR2_, and XB_Trap_) (Figure 1C). Here, the trap state XB_Trap_ is added to our previous model (Yoneda et al., 2021) to reproduce the stretch activation. Ca^2+^ sensitivity is reproduced based on state transitions in the T/T units on the thin filament (Figure 1D). The coefficients k_np_ and k_pn_ in the rate constants between the non-binding state N_XB_ and the weakly binding state P_XB_ are changed according to the state of the T/T unit above the myosin molecule. Cooperativity in nearest neighbor interactions is incorporated with the factors γ^ng^ and *γ* ^−ng^ to reproduce the force–pCa^2+^ relationship (Rice et al., 2003), where *γ* = 80 is used and *ng* = 0, 1, or 2 is the number of neighboring myosin molecules either in the weakly binding (P_XB_) or strong binding (XB_PreR_, XB_PostR1_, XB_PostR2_, and XB_Trap_) states.

**FIGURE 2:**
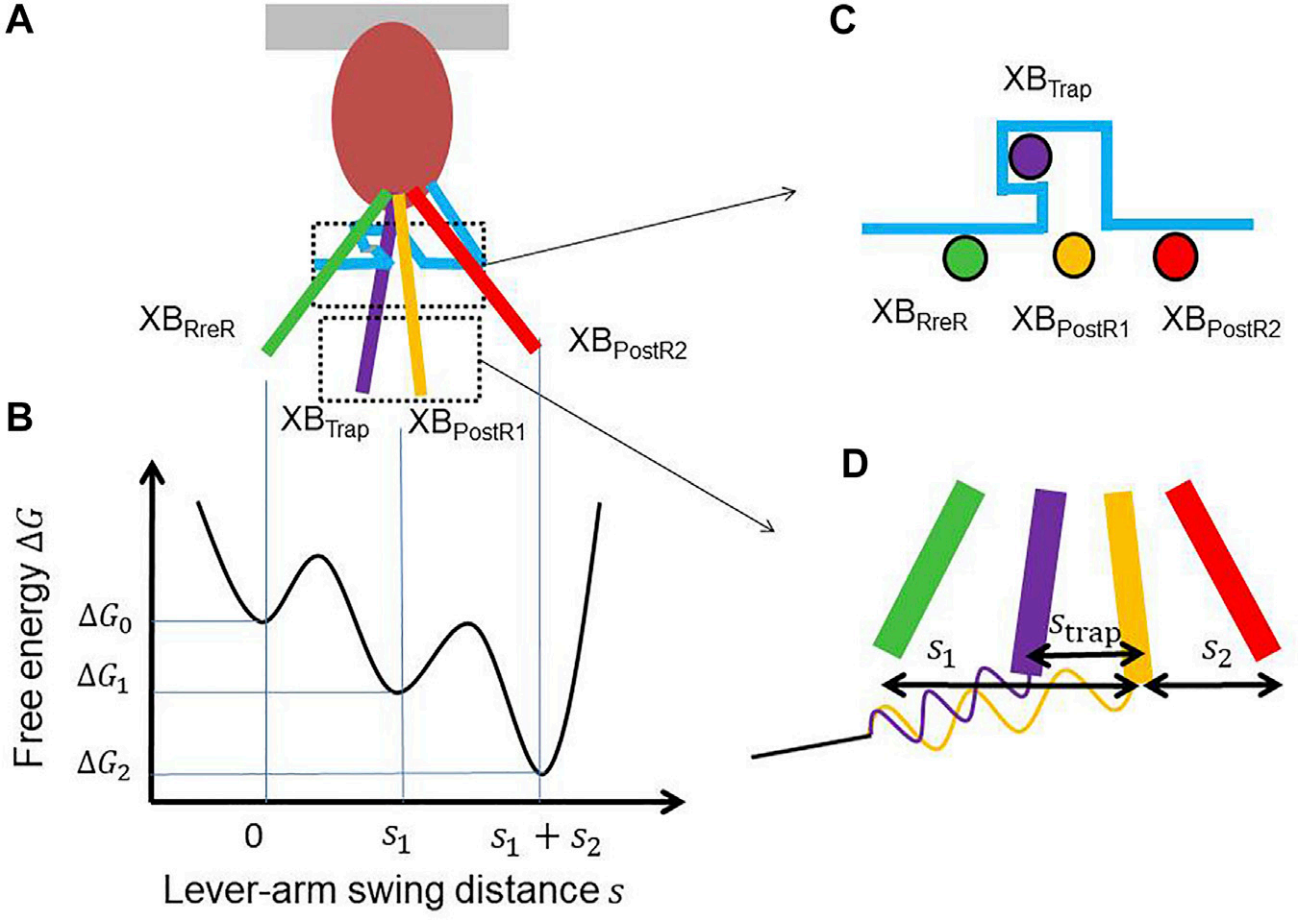
**(A)** Lever arm rotations at the four attached states with a hook (light blue) that traps the XB_Trap_ state. **(B)** The free energy profile that drives the power strokes. **(C)** A cross-sectional view of the lever arm positions of the four attached states. **(D)** Magnification of the lever arm rotations with the spring of the myosin rod. The distortion of the myosin rod decreases by $s_{trap}$ during trapping, while it increases by $s_{trap}$ while escaping.

The contraction force is generated by power stroke transitions in which the lever arm swing distance increases by $s_{1}$ and $s_{2}$ in the first and second strokes, respectively (Figures 2A,B). This increase in swing distance is directly reflected by an increase in myosin rod distortion (Figure 1B). In our model, the rate constants of the power and reverse strokes are given by functions of the myosin rod distortion x such that the Boltzmann equilibrium condition is fulfilled:

$$R_{i}\left(x\right)\equiv \frac{r_{f,i}\left(x\right)}{r_{b,i}\left(x+s_{i}\right)}=\text{exp}\left(\frac{W_{\text{rod}}\left(x\right)+\Delta G_{i-1}-W_{\text{rod}}\left(x+s_{i}\right)-\Delta G_{i}}{k_{B}T}\right),$$ (1)

where $k_{B}$ and T denote the Boltzmann constant and temperature, respectively. $\Delta G_{0}$ , $\Delta G_{1}$ , and $\Delta G_{2}$ are the free energies, respectively, at XB_PreR_, XB_PostR1_, and XB_PostR2_ (Figure 2B). $W_{\text{rod}}$ is the strain energy of the myosin rod. It should be noted that the rate constants are defined as a function of distortion at the origin of the transition. With the power stroke, the free energy decrease $\Delta G_{i-1}-\Delta G_{i}$ is transferred to an increase in strain energy $W_{\text{rod}}\left(x+s_{i}\right)-W_{\text{rod}}\left(x\right)$ . The strain energy is used for the external work *via* the half-sarcomere shortening, which corresponds to muscle shortening in the fiber direction.

Under the above formulation, the ratio $R_{i}$ increases when the distortion x decreases as the sarcomere shortens. Conversely, sarcomere lengthening (as in stretch activation) causes an increase in distortion x , resulting in a facilitation of reverse strokes. The chain reaction of reverse strokes and sarcomere lengthening is assumed to enable quick relaxation at the early diastole in the cardiac cycle (Washio et al., 2019; Hwang et al., 2021). However, the synchrony of this relaxation over the entire ventricular wall is needed to explain the realistic rapid decline in the left ventricular pressure. In our previous work (Washio et al., 2018), we introduced a trapping mechanism at the XB_PostR1_ state in the Langevin dynamics model to indicate the role of the stretch activation mechanism in the synchrony of relaxation. In the Langevin model, the potential that drove the power stroke was represented as a function of two parameters: the lever arm rotation distance, y , and the degree of lever arm deflection, θ . With these parameters, the total distance of the power stroke is given by s=y−θ . Here, the deflection θ increases as the pulling force imposed on the lever arm increases, and a high barrier in the y-direction is assumed for the large deflection θ in the potential landscape between XB_PreR_ and XB_PostR1_. In this way, reverse strokes from the XB_PostR1_ state to the XB_PreR_ state are prevented when the force is large. Thus, the myosin molecules in the XB_PostR1_ state with a large force are trapped. In this study, we introduce a similar trapping mechanism in our MC model based on a thermodynamically consistent formulation.

In Figure 2C, the trap mechanism is illustrated using a hook that inhibits the reverse transition to the XB_PreR_ state. The power stroke distance is slightly shortened by $s_{\text{trap}}$ in the trapping transition from XB_PostR1_ to XB_Trap_, while the system returns to the XB_PostR1_ state in the escaping transition from the XB_Trap_ state (Figure 2D). Thus, the ratio of the transition rates is given by:

$$T\left(x\right)\equiv \frac{r_{\text{escape}}\left(x\right)}{r_{\text{trap}}\left(x+s_{\text{trap}}\right)}=\exp\left(\frac{\Delta G_{\text{trap}}+W_{rod}\left(x\right)-\Delta G_{1}-W_{rod}\left(x+s_{\text{trap}}\right)}{k_{B}T}\right),$$ (2)

where $\text{Δ}G_{\text{trap}}$ is the free energy in the XB_Trap_ state. In this study, the rate constant for trapping is:

$$r_{\text{trap}}\left(x\right)=h_{\text{trap}}$$ (3)

and the rate constant for escaping is:

$$r_{\text{escape}}\left(x\right)=h_{\text{escape}}\exp\left(\frac{W_{rod}\left(x\right)-W_{rod}\left(x+s_{\text{trap}}\right)}{k_{B}T}\right).$$ (4)

From Eq. 2, the relationship between the ratio of the coefficients $h_{\text{trap}}\;\text{and}\;h_{\text{escape}}$ and the free energy difference is given by:

$$\frac{h_{\text{escape}}}{h_{\text{trap}}}=\exp\left(\frac{\text{Δ}G_{\text{trap}}-\text{Δ}G_{1}}{k_{B}T}\right).$$ (5)

If we assume linear elasticity for the myosin rod, the potential energy difference in Eq. 4 is represented by:

$$W_{rod}\left(x\right)-W_{rod}\left(x+s_{\text{trap}}\right)=-k_{\text{xb}}s_{\text{trap}}\left(x+\frac{1}{2}s_{\text{trap}}\right),$$ (6)

where $k_{\text{xb}}$ is the spring constant of the myosin rod. Eqs 4 and 6 indicate that as the distortion x increases, the myosin molecule becomes less likely to escape from the XB_Trap_ state. Thus, the half-sarcomere lengthening (as in stretch activation) implies a rapid increase in distortion x , resulting in trapping in the XB_Trap_ state. Eqs 4 and 6 also indicate that the degree of trapping depends on the loss of the power stroke distance $s_{\text{trap}}$ . Thus, the parameter $s_{\text{trap}}$ has the potential to reproduce stretch activation phenomena in various muscle types (Supplementary Figures S1,S2 for the plots of the rate functions).

**FIGURE 3:**
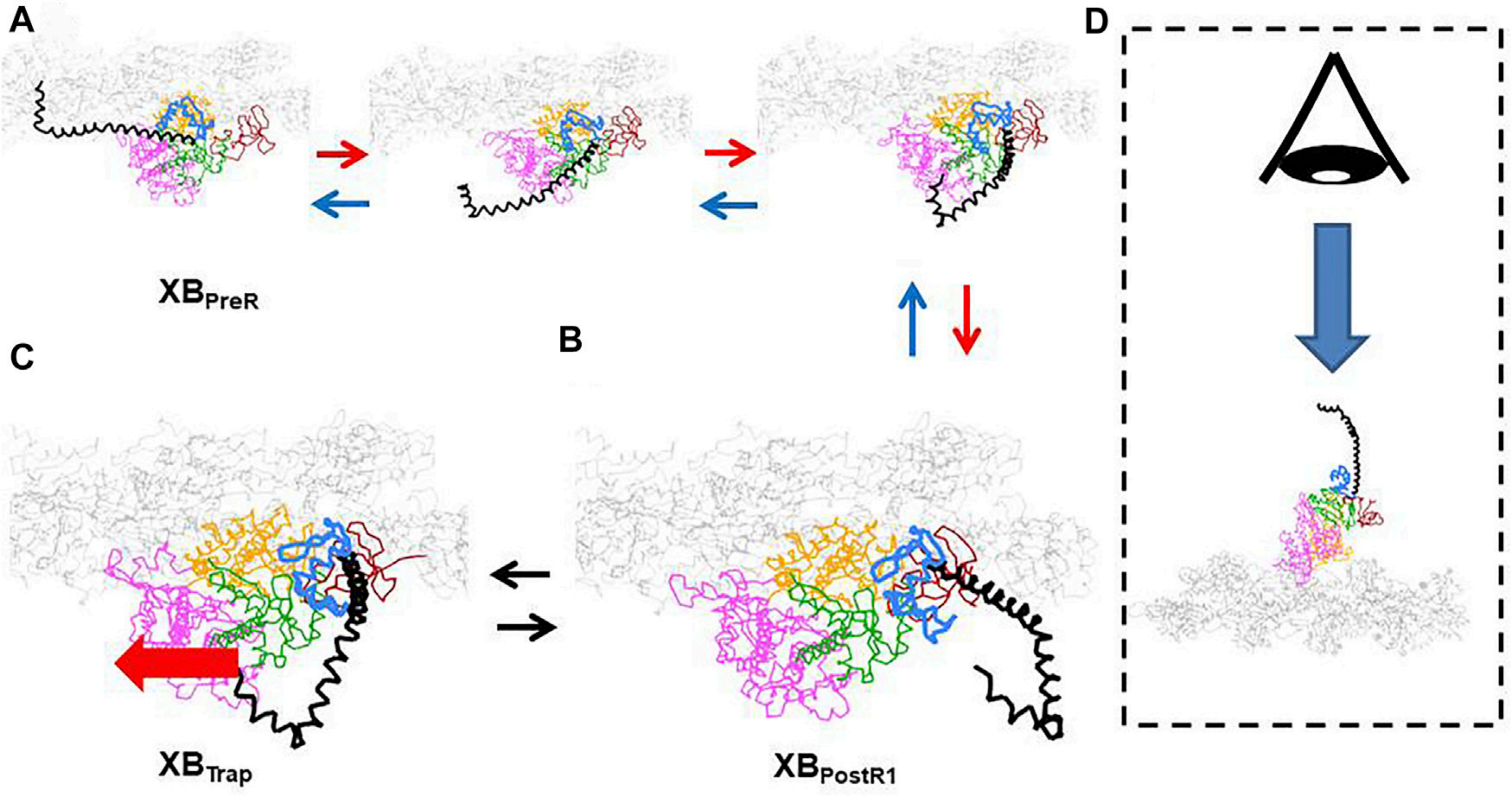
The conformation change of the myosin molecule in the first power stroke transition. **(A)** Intermediate conformations between the XB_PreR_ and XB_PostR1_ states. **(B)** Conformation in the XB_PostR1_ states **(C)** A candidate of conformation of the XB_Trap_ state with the imposed pulling force (thick red arrow). **(D)** The viewpoint for **(A–C)**. The myosin molecule is divided into the following domains: lever arm (black), converter (light blue), N-terminal (brown), central domain (green), upper 50 k (pink), and lower 50 k (orange). The thin filament is colored gray. In the XB_Trap_ state, the lever arm is trapped in the V-shaped region of the converter. The rotation of the converter might be inhibited in the case of a strong pulling force.

The structural mechanism of trapping has not yet been identified. However, the conformation change between the XB_PreR_ state and XB_PostR1_ state (Figure 3A) and the arrangement of the converter domain and the lever arm in the XB_PostR1_ state (Figure 3B) produced by our coarse-grained molecular dynamic simulation (Washio et al., 2021) indicate that the V-shaped region in the converter domain could hold the lever arm when it is strongly pulled in the opposite direction (Figure 3C). Note that these configurations of the myosin molecule are drawn from the viewpoint indicated in Figure 3D. Once the lever arm is strongly held in the V-shaped region, it might inhibit reverse rotation of the converter domain (from Figures 3A,B), resulting in trapping of the lever arm. Supplementary Video 1 shows a simulation of the power stroke transition from the XB_PreR_ state to the XB_PostR1_ state.

Determining which part of the contractile proteins is responsible for stretch activation remains controversial. Campbell and Chandra (Campbell and Chandra. 2006) reproduced stretch activation in cardiac muscle (Stelzer et al., 2006) by applying a numerical model, where they assumed that the thin filament regulatory unit (RU) was responsible. Conversely, Straight et al. (2019) proposed a myosin-based mechanism focusing on the ADP state, which corresponds to XB_PostR1_ in our MC model, although a trapping mechanism was not introduced in their model. A unique feature of our numerical model is its theoretical basement based on the Boltzmann distribution law under the strain energy for distortion of the myosin rod Eq. 2.

## Materials and methods

**FIGURE 4:**
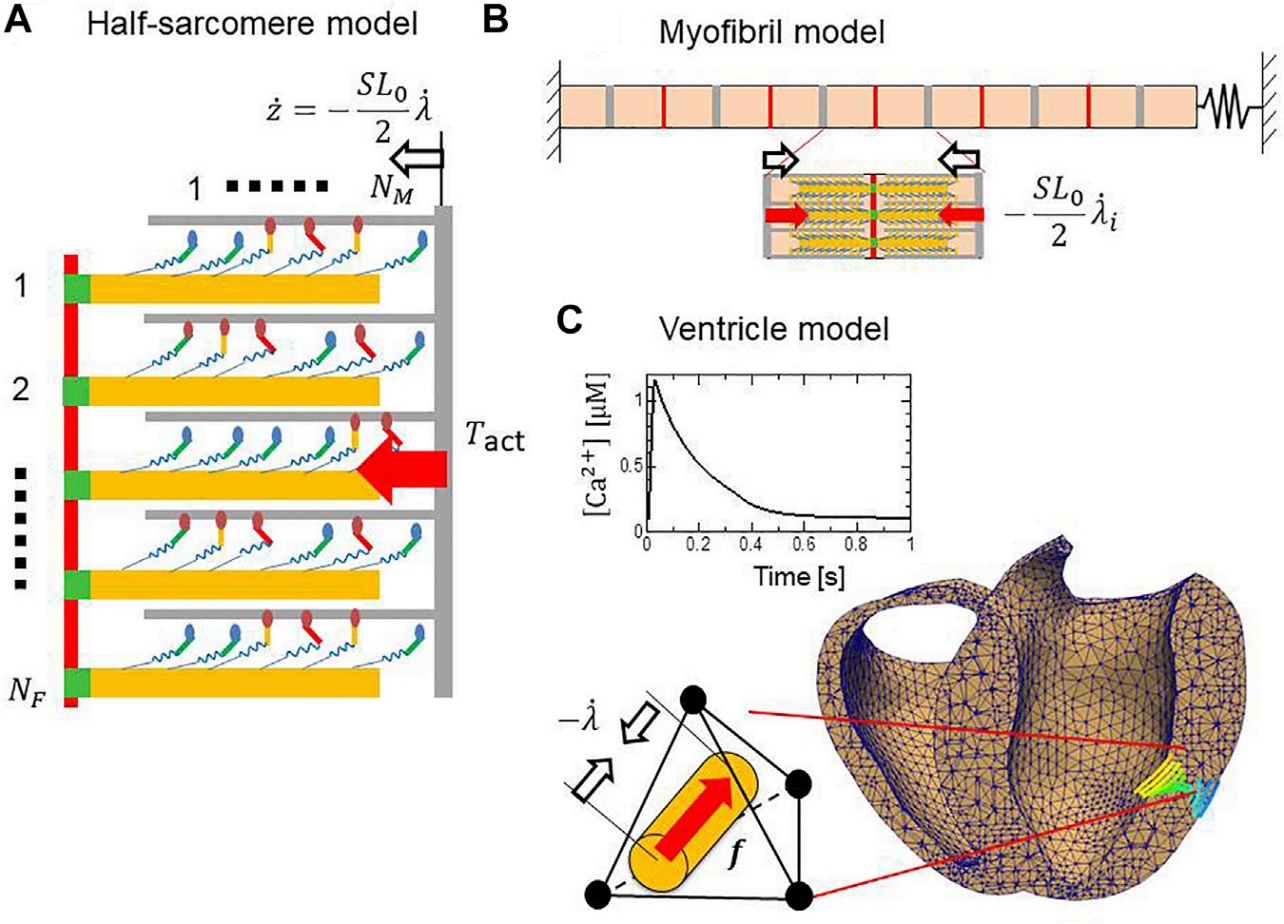
Computational models at three scales. **(A)** A half-sarcomere model consisting of $N_{F}$ filament pairs. In the stretch activation test, a rapid length change was imposed after the isometric force had matured. **(B)** The myofibril model for the SPOC simulation. The half-sarcomere models were imbedded to compute the active tensions in individual half-sarcomeres, whereas their stretches provide feedback to the half-sarcomere models. **(C)** A cross-section of the finite element bi-ventricular model and the transmural change in fiber orientation. The half-sarcomere models were imbedded in tetrahedral elements to compute the contraction force in the fiber direction, whereas the stretches in the fiber direction provided feedback to the half-sarcomere models. The boxed inset shows the Ca^2+^ transient given in each element.

Here, we introduce the cross-bridge MC model and its use in multiscale analyses. Cross-bridge MC models are arranged on a thick filament and interact with a thin filament. The pairs of filaments compose the half-sarcomere model (Figure 4A). Half-sarcomere models are imbedded into the myofibril model (Figure 4B) or the ventricle model (Figure 4C), where interactions of the half-sarcomere models in adjacent elements are analyzed. In the following, informative numerical results, representing basic properties of the MC cross-bridge model with the trapping mechanism are introduced to help readers understand the definitions of crucial parameters in the MC cross-bridge model.

The parameter values, which are not related to the trap model, are listed in Table 1. Some of these values came from the following references [H2021]: Hwang et al., 2021 [K2016]: Kolb et al., 2016 [L2000]: Lodish et al., 2000 [R2008]: Rice et al., 2008 [S2013]: Sato et al., 2013, and [Y2021]: Yoneda et al., 2021.

**TABLE 1:** Parameters for the actomyosin dynamics. “Adjusted” indicates that they were adjusted to reproduce the phenomena.

Parameter	Value	Unit	References	Parameter	Value	Unit	References
ATP hydrolysis energy				Sarcomere geometry			
$G_{\text{ATP}}$	76.5	pN⋅nm	[Y2021]	$SL_{0}$	1.9	μ m	[R2008]
$k_{B}T$	4.28	pN⋅nm	T=310	LM	1.65	μ m	[L2000]
**Stroke size and free energy** T=310				LB	0.16	μ m	[K2016]
$s_{1}$	6.0	nm	[H2021]	LA	1.0	μ m	[K2016]
$s_{2}$	4.0	Nm	[H2021]	$SA_{0}$	693	${\text{nm}}^{2}$	[S2013]
$\Delta G_{wb}$	$G_{\text{ATP}}$	pN/nm	Adjusted	**Force regulation through T/T unit**			
$\Delta G_{0}$	$G_{\text{ATP}}$	pN/nm	Adjusted	$K_{on}^{\prime }$	100	1/s	[Y2021]
$\Delta G_{1}$	0.7 $G_{ATP}$	pN/nm	Adjusted	$K_{off}^{\prime }$	30	1/s	[Y2021]
$\Delta G_{2}$	0	pN/nm	Adjusted	$K_{on}$	100	1/s	[Y2021]
—	—	—	—	$K_{off}$	150	1/s	[Y2021]
**Rod distortion energy** $W_{rod}$				$K_{basic}\;$	30	1/s	[Y2021]
$k_{xb}$	2.8	pN/nm	[K2010]	$Q_{\;}$	1.6	unitless	[Y2021]
$b_{xb}$	0.05	Unitless	[K2010]	μ	15	unitless	[Y2021]
$\xi_{1}$	4.35	Nm	[K2010]	γ	80	unitless	[R2003]
—				**Number of elements in a sarcomere**			
**Power stroke transition**				$N_{M}$	38	Unitless	[Y2021]
${\bar{r}}_{f,1}$ ${\bar{r}}_{f,2}$	10,000	1/s	Adjusted	$N_{T}$	32	umitles	[Y2021]
${\bar{r}}_{b,1}$ ${\bar{r}}_{b,2}$	1,000	1/s	Adjusted	—	—	—	—
$h_{1}$	3	1/s	Adjusted	—	—	—	—
**Attachment and detachment transition**				—	—	—	—
$c_{\text{pre}}$	3,000	1/s	adjusted	—	—	—	—
$r_{NXB}$	225	1/s	adjusted	—	—	—	—

### Parameters for the trap mechanism

Three parameters, $s_{\text{trap}}$ , $h_{\text{trap}}$ , and $h_{\text{escape}}$ , characterize the trapping. We adjusted these parameters so that the experimental stretch activation results reported by Stelzer et al. (2006) were reproduced. The adjusted parameter values are $s_{\text{trap}}$ = 1.3 nm, $h_{\text{trap}}$ = 50 1/s, and $h_{\text{escape}}$ = 5,000 1/s. From Eq. 5, we find that $\text{Δ}G_{\text{trap}}-\text{Δ}G_{1}$ = 4.6 $k_{B}T$ . According to Eq. 6, the rate constants of trapping $r_{\text{trap}}$ and escaping $r_{\text{escape}}$ are equal at a distortion of x = 4.8 nm in the XB_Trap_ state and $x+s_{\text{trap}}$ = 6.1 nm in the XB_PostR1_ state, assuming a spring constant of $k_{\text{xb}}$ = 2.8 pN/nm (Kaya and Higuchi, 2010) and a physiological body temperature of T = 310 K.

### Control model of attachment and detachment and its effects on heart pumping

In our model, we assume that attachment, which represents the transition from the P_XB_ state to the XB_PreR_ state (Figure 1C), is allowed only in the single overlap region of the thin and thick filaments. The myosin head (MH) (# i ) is situated in the single overlapping region only if the following condition is fulfilled:

$$\text{max}\left(LA-\frac{SL}{2},\frac{SL}{2}-LA\right)\leq \frac{LB}{2}+\frac{2\left(i-0.5\right)}{n_{M}\left(LM-LB\right)}\leq \frac{SL}{2}\;.$$ (7)

Here, the middle term is the distance from the center of the sarcomere . LM, LB, and LA represent the lengths of the thick filament, bare zone, and thin filament, respectively (Figure 1B). SL is the sarcomere length. The parameters for the sarcomere geometry were determined from cardiac sarcomeres (Rodriguez et al., 1993; Lodish et al., 2000; Rice et al., 2008; Kolb et al., 2016).

Two states (Ca-off and Ca-on) are assumed by each T/T unit (Figure 1D). The transitions between the states of the T/T unit are determined by the Ca^2+^ concentration, $\left[{\text{Ca}}^{2+}\right]$ , and the two parameters ${\bar{K}}_{\mathrm{on}}$ and ${\bar{K}}_{\mathrm{off}}$ . Those two parameters are defined as follows:

$${\bar{K}}_{\text{on}}=\{\begin{array}{c} K_{\text{on}}^{\prime }\;\;\;\;\;\;if\;there\;is\;an\;MH\;binding\;below,\\ K_{\text{on}}\;otherwise. \end{array}$$

$${\bar{K}}_{\text{off}}=\{\begin{array}{c} K_{\text{off}}^{\prime }\;\;\;\;\;\;\;\;if\;there\;is\;an\;MH\;binding\;below,\\ K_{\text{off}}\;\;\;otherwise. \end{array}$$

The transitions between the N_XB_ and P_XB_ states (Figure 1C) are affected by the status of the T/T unit above, *via* modifications of $k_{\mathrm{np}}$ and $k_{\mathrm{pn}}$ , as well as by the states of the neighboring MHs through the integer ng . The value of ng (=0, 1, or 2) represents the number of neighboring MHs in the P_XB_ state or the four attached states. The corresponding T/T unit index τ for the i -th MH is given by:

$$\text{τ}=\lfloor \frac{0.5LB+\left(i-0.5\right)S_{M}-\left(SL/2-LA\right)}{S_{T}}\rfloor .$$ (8)

Here, ⌊ ⌋ indicates the floor function, which rounds down after the decimal point. The parameter $S_{M}=0.5\left(LM-LB\right)/N_{M}$ represents the spacing of the MHs, and $S_{T}=LA/N_{T}$ is the spacing of the T/T units. The corresponding T/T unit exists only if $1\leq \text{τ}\leq N_{T}$ . Based on this correspondence, the factors $k_{\mathrm{np}}$ and $k_{\mathrm{pn}}$ of the rate constants are given by:

$$k_{np}=\{\begin{array}{c} \delta_{OV}K_{\text{np}1}\;\;\;\;\;if\;the\;T/T\;unit\;above\;is\;in\;the\;Ca-on\;state,\\ \delta_{OV}K_{\text{np}0}\;\;\;\;\;otherwise. \end{array}$$ (9)

$$k_{pn}=\{\begin{array}{c} K_{pn1}\;\;\;\;\;\;if\;the\;T/T\;unit\;above\;is\;in\;the\;Ca-on\;state,\\ K_{\mathrm{pn}0}\;\;\;\;\;otherwise. \end{array}$$ (10)

**FIGURE 5:**
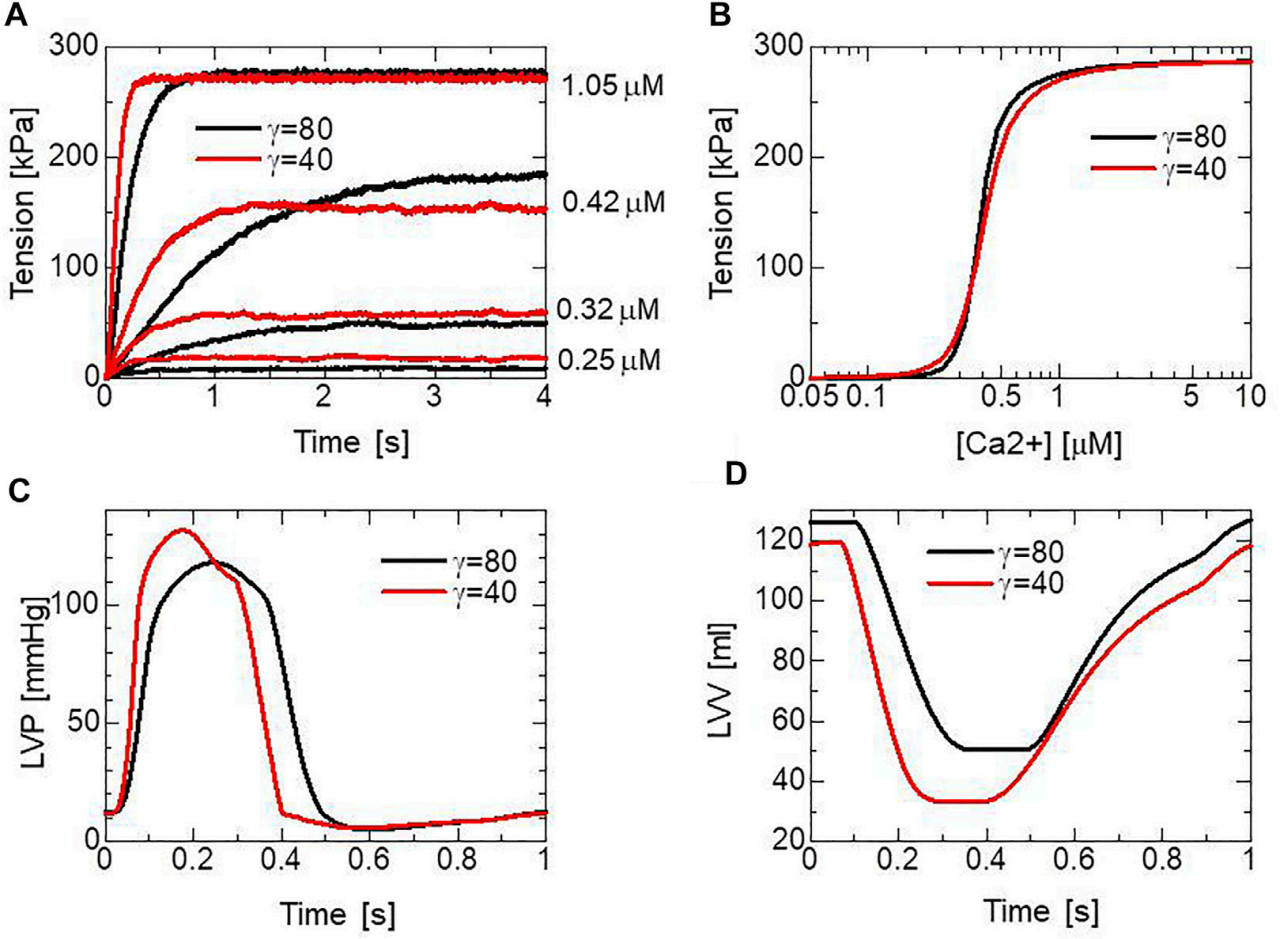
The simulated pCa-tension relationship of the MC cross-bridge model and its effects on heart pumping. **(A)** Time courses of the active tensions in the isometric contraction for [Ca^2+^] = 0.25, 0.42, and 1.05 μM with γ = 80 (black) and γ = 40 (red). **(B)** Isometric tensions in the equilibrium states averaged over the time interval [3.5 s, 4 s] with γ = 80 (black) and γ = 40 (red). The half-sarcomere length was fixed at 1.14 μm ( λ=1.2 ). **(C)** Time transients of left ventricular pressures (LVP) with γ = 80 (black) and γ = 40 (red). **(D)** Time transients of the left ventricular volume (LVV) with γ = 80 (black) and γ = 40 (red).

Here, $\delta_{OV}=1$ if the MH is located in the single overlapping region with the thin filament; otherwise, $\delta_{OV}=0$ . This, along with $\gamma^{ng}$ or $\gamma^{-ng}$ (γ=80) , represents the nearest-neighbor cooperativity of the MHs, following Rice et al. (Rice et al., 2003), which plays an important role in the force-pCa relationship, as shown in Figures 5A,B. From the cooperativity with γ=80 , the cardiac muscle in our ventricle model can relax in the diastole when [Ca^2+^] is lower than 0.1 μM (Figure 4C), whereas the diastole deteriorates slightly with γ=40 (Figures 5C,D). The times to reach equilibria differ substantially and are dependent on the Ca^2+^ concentration and parameter γ . Thus, cooperativity also affects the maximal rising rate of the left ventricular pressure (dP/dt_max_), which is an important contractility index used in the medical community (Figures 5C,D).

We assume that one thin filament in the three-dimensional arrangement corresponds to two thin filaments in our half-sarcomere model. This case arises because we assumed that cooperative behavior exists along the tropomyosin molecules wrapped around the thin filament in a double-spiral fashion, and one spiral is modeled in our half-sarcomere. The constants $K_{\mathrm{np}0}$ , $K_{\mathrm{np}1}$ , $K_{\mathrm{pn}0}$ , and $\;K_{\mathrm{pn}1}$ are determined from Q , $K_{\mathrm{basic}}$ , and μ , as follows:

$$K_{np0}=F_{K}\left(SL\right)\frac{QK_{basic}}{\mu },\;K_{np1}=F_{K}\left(SL\right)QK_{basic},\;K_{pn0}=K_{pn1}=K_{basic}\gamma^{2}.$$ (11)

Here, μ>1 controls the degree of cross-bridge inhibition for the T/T units in states other than Ca-on, and Q controls the ratio of binding states for the MHs. To reproduce the SL dependence in the active contraction tension, the following function $F_{K}\left(SL\right)$ is multiplied to define $K_{\mathrm{np}0}$ and $K_{\mathrm{np}1}$ .

$$F_{K}\left(SL\right)=\{\begin{array}{c} 1,\;\;\;\;\;\;\;\;\;\;\;\;\;\;\;\;\;\;\;\;\;\;\;\;\;\;\;\;\;\;\;\;\;\;\;\;\;\;SL\geq SL_{Q},\\ 1-\alpha_{Q}\left(SL_{Q}-SL\right),\;\;\;\;SL<SL_{Q}. \end{array}$$ (12)

The values $\alpha_{Q}$ = 0.25 [1/ μ m] and $SL_{Q}$ = 2.1 μ m are used in this study.

The rate constants of attachment $r_{a}\;$ and detachment $r_{d}\;$ between the P_XB_ state and XB_PreR_ state are based on the assumed free energies, $\Delta G_{wb}\;$ and $\Delta G_{0},$ of the P_XB_ state and XB_PreR_ state, respectively:

$$r_{a}=c_{\text{pre}}\text{exp}\left(-\frac{\Delta G_{0}-\Delta G_{wb}}{k_{B}T}\right),\;\;\;r_{d}=c_{\text{pre}}.$$ (13)

The initial rod distortion at attachment is given stochastically based on a Boltzmann distribution determined from the rod strain energy, $W_{rod}\left(x\right)$ (Washio et al., 2016). The detachment rate constant for transitions from the XB_PostR2_ state to the N_XB_ state is $r_{\text{NXB}}$ .

Using the rate constants, we also considered forced detachments from the XB_PreR_, XB_PostR1_, and XB_PostR2_ states to the N_XB_ state caused by extreme strain on the myosin rod:

$$d_{XB,i}\left(x\right)=\{\begin{array}{c} 0,\;\;\;\;\;\;\;\;\;\;\;\;\;\;\;\;\;\;\;\;\;\;\;\;\;\;\;\;\;\;\;\;\;\;\;\;\;\;\;\;\;\;\;\;\;\;\;\;\;\;\;x<x_{XB,i},\\ c_{XB,i}\left(\exp\left(a_{XB,i}\left(x-x_{XB,i}\right)\right)-1\right),x\geq x_{XB,i}, \end{array}\;\;\;\;\;\;\;i=0,\;1,\;2.$$ (14)

Similarly, the rate constant of detachment from the XB_Trap_ state is given by:

$$d_{\text{trap}}\left(x\right)=\{\begin{array}{c} 0,\;\;\;\;\;\;\;\;\;\;\;\;\;\;\;\;\;\;\;\;\;\;\;\;\;\;\;\;\;\;\;\;\;\;\;\;\;\;\;\;\;\;\;\;\;\;\;\;x<x_{XB,t},\\ c_{XB,t}\left(\exp\left(a_{XB,t}\left(x-x_{XB,t}\right)\right)-1\right),\;\;\;\;\;x\geq x_{XB,t}. \end{array}$$ (15)

We paid close attention to the adjustments of parameters in Eqs 14 and 15 in reproducing the stretch activation. These parameters affect the degree of the increased delayed force. In this study, we used the following parameters to reproduce the characteristics of cardiac muscles: $c_{XB,0}$ = 100 1/s, $c_{XB,1}$ = $c_{XB,2}$ = $c_{XB,t}$ = 20 1/s, $a_{XB,0}$ = 1 1/nm, $a_{XB,1}$ = $a_{XB,2}$ = $a_{XB,t}$ = 0.1 1/nm, $x_{XB,0}$ = 3 nm, $x_{XB,1}$ = 8.5 nm, $x_{XB,2}$ = 13 nm, $x_{XB,t}$ = 8.5 nm.

### Power stroke and reverse transitions

In our model, the rate constants of the power and reverse strokes are determined from the Kramers escape theory (Kramers 1940), in which rate constants are defined by the Boltzmann factor associated with the height of the energy barrier from the origin.

$${\hat{r}}_{f,i}\left(x\right)=h_{i}exp\left(\frac{\Delta G_{i-1}+W_{rod}\left(x\right)-W_{rod}\left(x+s_{i}/2\right)}{k_{B}T}\right),$$ (16)

$${\hat{r}}_{b,i}\left(x+s_{i}\right)=h_{i}exp\left(\frac{\Delta G_{i}+W_{rod}\left(x+s_{i}\right)-W_{rod}\left(x+s_{i}/2\right)}{k_{B}T}\right).$$ (17)

Here, $W_{rod}\left(x+s_{i}/2\right)$ is introduced because we assume that the barrier is given at the middle of the power stroke distances for the two states. In addition, we adopt upper limits to those transition rate constants because the power stroke transitions are thought to accompany a release of Pi and ADP from the nucleotide-binding pocket in the MH during the first and second strokes, respectively. Thus, the reverse stroke transitions must accompany a rebinding of these molecules. We assumed that the rates of chemical reactions for the release and rebinding are given by ${\bar{r}}_{f,i}$ and ${\bar{r}}_{b,i}$ , respectively. With these upper limits, the temporary rate constants are modified so that the Boltzmann equilibrium in Eq. 1 is preserved:

$$\;r_{f,i}\left(x\right)=\{\begin{array}{c} {\bar{r}}_{f,i},\;\;\;\;\;\;\;\;\;\;\;\;\;\;\;\;\;\;\;\;\;x\leq {\bar{x}}_{f,i}\\ {\hat{r}}_{f,i}\left(x\right),\;\;\;{\bar{x}}_{f,i}<x\leq \;{\bar{x}}_{b,i}\\ \frac{{\hat{r}}_{f,i}\left(x\right){\bar{r}}_{b,i}}{{\hat{r}}_{b,i}\left(x+s_{i}\right)},\;\;\;\;\;\;\;\;x>{\bar{x}}_{b,i} \end{array},$$ (18)

$$r_{b,i}\left(x+s_{i}\right)=\{\begin{array}{c} \frac{{\hat{r}}_{b,i}\left(x+s_{i}\right){\bar{r}}_{f,i}}{{\hat{r}}_{f,i}\left(x\right)},\;\;\;\;\;\;\;\;\;\;\;\;\;\;\;\;\;x\leq {\bar{x}}_{f,i}\\ {\hat{r}}_{b,i}\left(x+s_{i}\right),\;\;{\bar{x}}_{f,i}<x\leq \;{\bar{x}}_{b,i}\\ {\bar{r}}_{b,i},\;\;\;\;\;\;\;\;\;\;\;\;\;\;\;\;\;\;\;\;\;\;\;\;\;\;\;\;\;\;\;\;\;\;\;\;\;\;\;\;\;\;x>{\bar{x}}_{b,i} \end{array}.$$ (19)

Here, ${\bar{x}}_{f,i}$ and ${\bar{x}}_{b,i}$ are the distortions at which the temporary rates reach the upper limits ( ${\hat{r}}_{f,i}\left({\bar{x}}_{f,i}\right)={\bar{r}}_{f,i},\;{\hat{r}}_{b,i}\left({\bar{x}}_{b,i}+s_{i}\right)={\bar{r}}_{b,i}\;$ ).

The elastic force of a myosin rod is nonlinear with respect to the strain (Kaya and Higuchi, 2010). We assume that the myosin rod behaves as a linear spring for positive stretches, whereas nonlinear behavior is introduced for negative stretches because of the slack induced along the myosin rod. The strain energy $W_{rod}$ is found by integrating the force $F_{\text{rod}}$ from x=0 , defined by:

$$F_{rod}\left(x\right)=\{\begin{array}{c} b_{xb}k_{xb}\left(x+\xi_{1}\right)-F_{1},\;\;\;\;\;\;\;x<-\xi_{1},\\ \frac{k_{xb}}{a_{xb}}\left(\exp\left(a_{xb}x\right)-1\right),\;\;\;\;\;\;\;-\xi_{1}\leq x<0,\\ k_{xb}x,\;\;\;\;\;\;\;0\leq x. \end{array}$$ (20)

Here, $a_{xb}\;$ and $F_{1}$ are determined from the other parameters, so that the function $F_{rod}$ and its first derivative are continuous at ξ=0 and $-\xi_{1}$ :

$$\{\begin{array}{c} a_{xb}=-\frac{\left(\ln b_{xb}\right)}{\xi_{1}},\;\;\;\;\;\;\;\;\;\;\;\;\;\;\;\;\;\;\;\;\\ F_{1}=\frac{k_{xb}\left(1-\exp\left(-a_{xb}\xi_{1}\right)\right)}{a_{xb}}. \end{array}$$ (21)

### Half-sarcomere model and its basic properties

We constructed a half-sarcomere model (Figure 5A) with $n_{F}$ one-dimensional filament pairs in which the active tension generated by the bound myosin molecules is given as:

$$T_{act}=\frac{2R_{S}}{SA_{0}\cdot n_{F}}\overset{n_{F}}{\underset{\beta =1}{\sum }}\overset{n_{M}}{\underset{\alpha =1}{\sum }}\delta_{A,\alpha ,\beta }\frac{\text{d}W_{rod}\left(x_{\alpha ,\beta }\right)}{\text{d}x}\;.$$ (22)

Here, $\delta_{A,\alpha ,\beta }=1$ if the MH is in an attached state; otherwise, $\delta_{A,\alpha ,\beta }=0$ . The parameter $SA_{0}$ is the cross-sectional area per thin filament in an unloaded half-sarcomere (Sato et al., 2013). The factor of 2 comes from the fact that our one-dimensional model corresponds to one of the double spirals of actin monomers along the thin filament. The parameter $R_{S}$ is the sarcomere volume ratio under the unloaded condition. $R_{S}$ = 0.5 is used, which indicates that 50% of the total volume is occupied by the sarcomere in the cardiac muscle.

For the feedback between sarcomere dynamics and actomyosin dynamics, the relation between the time transients of the molecular and sarcomere variables can be expressed by:

$${\;}^{\text{t}}x{=}^{t}{\;}^{\text{A}}x-({\;}^{t}z-{\;}^{t}{\;}^{\text{A}}z).$$ (23)

Here, $t_{A}$ is the most recent time at which the myosin molecule was attached, x tA is the initial distortion at the attachment, and z t is the half-sarcomere shortening length from the unloaded condition:

$${\;}^{\text{t}}z=-\frac{SL_{0}}{2}\left({\;}^{\text{t}}\lambda -1\right),$$ (24)

where $SL_{0}$ is the unloaded SL. Here, the stretch λ t is the parameter given by macroscale models. The initial distortion is given from the Boltzmann distribution, assuming a fluctuation in the MH position under the potential $W_{rod}$ (Washio et al., 2016).

**FIGURE 6:**
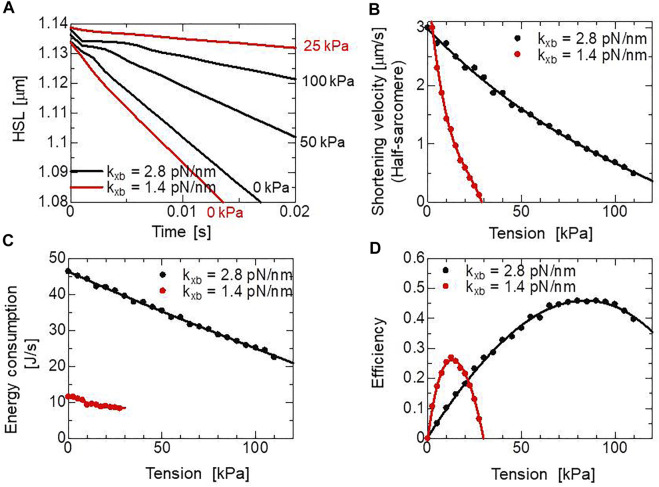
Simulated half-sarcomere length changes under isotonic conditions with the two cross-bridge model using different myosin rod stiffnesses, $k_{xb}$ = 1.4 pN/nm and $k_{xb}$ = 2.8 pN/nm. **(A)** Time courses of the half-sarcomere length under isotonic conditions with isotonic tensions of 0, 50, and 100 kPa for the cross-bridge model with $k_{xb}$ = 2.8 pN/nm, and 0, 25 kPa for the cross-bridge model with $k_{xb}$ = 1.4 pN/nm. **(B)** Half-sarcomere shortening velocities under isotonic tensions after the release starting from the half-sarcomere length at 1.14 μm ( λ=1.2 ). The velocities were calculated from changes of the half-sarcomere length from 1.12 to 1.09 after the release under isotonic conditions, where [Ca^2+^] was fixed at 0.4 μM . **(C)** The energy consumption rate per the left ventricular wall volume (157 ml) of the ventricle model. **(D)** Efficiencies for shortening under isotonic conditions.

The half-sarcomere shortening ( z˙ t>0 ) reduces the rod distortion (Eq. 23), which facilitates the power stroke (Eq. 16) and the deterioration of the reverse stroke (Eq. 17). Therefore, both the active tension and energy consumption are affected by the half-sarcomere shortening velocity. In Figure 6A, changes in the half-sarcomere length with various isotonic tensions computed using the MC cross-bridge model are depicted. In these isotonic contractions, the shortening velocities were computed over the common range of the half-sarcomere length (1.09–1.12 μm) to derive the tension-shortening velocity relationship (Figure 6B). By counting the number of detachments from the XB_PostR2_ state (Figure 1C: $r_{NXB}$ ), the energy consumption rates were evaluated assuming the detachment requires the energy $G_{ATP}=76.5\;\text{pN}\cdot \text{nm}$ , which is used in power stroke transitions (Figure 6C), as represented by Eq. 1. In the MC cross-bridge model, we assumed that 30% of $G_{ATP}$ is consumed during the first power stroke (6 nm), and the remaining $G_{ATP}$ is consumed during the second power stroke (4 nm) (See Table 1). The efficiencies were computed by dividing the work rates by the energy consumption rates (Figure 6D).

The energy loss in the cross-bridge cycle is given during the power stroke transitions and detachment from the XB_PostR2_ state. The difference between $\Delta G_{i}-\Delta G_{i+1}$ and $W_{rod}\left(x+s_{i}\right)-W_{rod}\left(x\right)$ is lost in the former, whereas the rod strain energy, $W_{rod}\left(x\right)$ , at the detachment is lost in the latter. To observe the influence of the stiffness parameter $k_{xb}$ in Eq. 20 on the dynamics in isotonic shortening, the cross-bridge performance was evaluated with the stiffness parameter $k_{xb}$ = 1.4 pN/nm, which is half of our parameter $k_{xb}$ = 2.8 pN/nm found by Kaya and Higuchi, 2010. In this case, the phase changes of the shortening velocity were not reproduced (Figure 6A), and the changes in the energy consumption rate for the tension is gentler than our model because the rate constants of power stoke transitions are less sensitive to the tension than the case of $k_{xb}$ = 2.8 pN/nm. As a result, the peak efficiency is approximately half for our case (Figure 6D). The strain energy after the second power stroke ( $0.5{\left(s_{1}+s_{s}\right)}^{2}k_{xb}$ ) with $k_{xb}$ = 1.4 pN/nm is 70 pN⋅nm assuming isometric contraction and zero distortion at XB_PreR_. This energy is almost equal to $G_{ATP}$ . However, rod distortion decreases by half-sarcomere shortening until reaching XB_PostR2_ (Eq. 23) during isotonic contraction. Therefore, 75% of the energy loss is accounted for even at the optimal shortening velocity.

### Coupling with macroscale models

In this study, coupling of the half-sarcomere model (Figure 4A) and the myofibril model (Figure 5B) or the ventricle model (Figure 4C) was achieved by applying the multiple-step active stiffness integration scheme in the exchange of the active tension $T_{act}$ and stretch λ between the two scales. The macroscale model is driven by the active stress given by the active tension in the half-sarcomere model, whereas the stretch in the fiber orientation in the macroscale model provides feedback to the length change of the half-sarcomere model.

In the macroscale model, a much larger time step ΔT=nΔt compared with the MC time step Δt is used to save computational time. For a given stretch ${\;}^{T+\Delta T}\lambda$ at T+ΔT , the macroscopic active tension $T_{\text{act},\left[T,T+\text{Δ}T\right]}$ over the time interval [T,T+ΔT] is determined by taking the time average of the active tension in the half-sarcomere model given for each MC step within an interval of Δt .

Tact,[T,T+ΔT](λ T+ΔT)=1n∑k=1n Tact(T+knΔT,λT+kn( T+ΔTλ−λT)). (25)

The active tension $T_{\text{act}}$ in the half-sarcomere model is given as a function of time and stretch for each MC step. The stretch for each MC step is determined by interpolating the stretches at T and T+ΔT to evaluate an appropriate stiffness of the binding myosin molecules in macroscopic Newton iterations. Note that the state transitions in the MC computations are calculated only once before the Newton iterations, assuming a stretch of $\lambda^{\;T+\Delta T}=\lambda^{T}+k\Delta \text{t}\lambda^{T}$ for each MC step. However, the rod distortion $\lambda^{\;T+\Delta T}$ is re-evaluated from Eqs 23 and 24 by replacing λ˙ T with $\left(\lambda^{\;T+\Delta T}{-}^{T}\lambda \right)/\Delta T$ using the updated stretch $\lambda^{\;T+K\Delta T}$ in the macroscopic Newton iterations. In this study, Δt = 2.5 μs is used for the MC step, while ΔT = 0.1 ms and ΔT = 1.25 ms are used, respectively, for the myofibril and ventricle models.

For simplicity, we introduce the Newton iteration for the one half-sarcomere model under the isotonic condition, as in Figure 6A.

$$\gamma_{L}\dot{\lambda }+\frac{d\varphi_{L}}{d\lambda }\left(\lambda \right)+T_{act}-T_{iso}=0,$$ (26)

where $\gamma_{L}=0.01\text{ kPa}\cdot \text{s}$ is the sarcomere friction, $\varphi_{L}$ is the deformation energy for stretching in the longitudinal direction (Washio et al., 2019), and $T_{iso}$ is the isotonic tension imposed on the cardiac muscle. Eq. 26 is discretized as:

$$\gamma_{L}\frac{{\;}^{T+\Delta T}\lambda {-}^{T}\lambda }{\Delta T}+\frac{d\varphi_{L}}{d\lambda }\left({\;}^{T+\Delta T}\lambda \right)+T_{\text{act},\left[T,T+\text{Δ}T\right]}\left({\;}^{T+\Delta T}\lambda \right)-T_{iso}=0.$$ (27)

After executing n -times MC steps in [T,T+ΔT] , where the half-sarcomere shortening length $z^{\;T+K\Delta T}$ at the *k*th step is given by assuming $\lambda^{\;T+K\Delta T}{=}^{T}\lambda +k\Delta {\text{t}}^{T}\dot{\lambda }$ with ${\;}^{T}\dot{\lambda }={(}^{T}\lambda {-}^{T-\Delta T}\lambda )/\Delta T$ , the initial solution of the Newton iteration is set as $\lambda^{\;T+K\Delta T}=\lambda^{T}$ , and the following linearized equation of Eq. 27 at ${\;}^{T+\Delta T}\lambda {=}^{T+\Delta T}\lambda^{\left(i\right)}$ is iteratively solved until the magnitude of the residual $R^{\left(i\right)}$ is reduced sufficiently.

$$\left(\frac{\gamma_{L}}{\Delta T}+\frac{d^{2}\varphi_{L}}{d\lambda^{2}}{(}^{T+\Delta T}\lambda^{\left(i\right)})+K_{\text{act},\left[T,T+\text{Δ}T\right]}{(}^{T+\Delta T}\lambda^{\left(i\right)})\right){(}^{T+\Delta T}\lambda^{\left(i+1\right)}{-}^{T+\Delta T}\lambda^{\left(i\right)})=R^{\left(i\right)},$$ (28)

where the residual is given by

R(i)=−(γL T+ΔTλ(i)−λ TΔT+dφLdλ( T+ΔTλ(i))+Tact,[T,T+ΔT]( T+ΔTλ(i))−Tiso). (29)

Here, $K_{\text{act},\left[T,T+\text{Δ}T\right]}\left({\;}^{T+\Delta T}\lambda \right)$ is the total stiffness of the binding myosin rods given as:

$$K_{\text{act},\left[T,T+\text{Δ}T\right]}\left({\;}^{T+\Delta T}\lambda \right)=\frac{dT_{\text{act},\left[T,T+\text{Δ}T\right]}}{d\lambda }\left({\;}^{T+\Delta T}\lambda \right).$$ (30)

See our previous work (Yoneda et al., 2021) for the actual computation of the right-hand side in Eq. 30. Under normal situations, the stiffness coefficient $K_{\text{act},\left[T,T+\text{Δ}T\right]}$ increases greatly to be much larger than $\gamma_{L}/\Delta T+d^{2}\varphi_{L}/d\lambda^{2}$ during contraction. Therefore, stiffness caused by the binding myosin rods must be correctly incorporated in the total stiffness, as in Eq. 28; otherwise, the time integration scheme generates inaccurate solutions without using a small time step ΔT in a similar order of magnitude to the MC time step Δt (Yoneda et al., 2021).

The above implicit time integration scheme can be naturally extended to more general cases where the half-sarcomere models are imbedded in different elements that interact with each other at the element interfaces. In the myofibril model (Figure 4B), two degrees of freedom, the stretches $\lambda_{i}$ and $\mu_{i}$ , respectively, in the longitudinal and transverse directions, are given to each half-sarcomere element (Washio et al., 2019). For each half-sarcomere, the passive deformation energy per unit volume is given by

$$\text{ψ}\left(\lambda ,\mu \right)=\varphi_{L}\left(\lambda \right)+\varphi_{T}\left(\mu \right)+\frac{1}{2}k_{LT}R_{LT}{\left(\lambda ,\mu \right)}^{2}.$$ (31)

Here, the function $\varphi_{T}$ is the deformation energy for the transverse stretch, and the last term is a weak penalty term associated with the inverse SL-lattice space (LS) relationship, which constrains the half-sarcomere deformation to make $\left|R_{LT}\left(\lambda ,\mu \right)\right|$ small. In our model, a simple linear relation:

$$R_{LT}\left(\lambda ,\mu \right)=\lambda -1+2\beta_{R}\left(\mu -1\right),$$ (32)

with $\beta_{R}=2$ is applied (Washio et al., 2019). Concerning the elasticity with differences in LS between adjacent half-sarcomeres, the following deformation energy per unit volume is further applied.

$${\text{φ}}_{TA,i}\left({\text{μ}}_{i},{\text{μ}}_{i+1}\right)=\{\begin{array}{c} k_{MM}{\left(\mu_{i}-\mu_{i+1}\right)}^{2},\;i=1,3,\cdots \\ k_{MZ}{\left(\mu_{i}-\mu_{i+1}\right)}^{2},i=2,4,\cdots \end{array}$$ (33)

where the half-sarcomeres are separated by the M-band for the top expression, and are separated by the Z-disc for the lower expression.

Within each half-sarcomere, the following tensions act at the left and right edges:

$$T_{SR,i}=\gamma_{L}{\dot{\lambda }}_{i}+\frac{d\varphi_{L}}{d\lambda }\left(\lambda_{i}\right)+k_{LT}R_{LT}\left(\lambda_{i},\mu_{i}\right)+T_{act,i}\;,\;i=1,\cdots ,ns.$$ (34)

The longitudinal mechanical equilibrium condition at the element boundaries:

$$T_{SR,i}-T_{SR,i+1}=0,$$ (35)

and the transversal mechanical equilibrium condition at each element:

$$\gamma_{T}{\dot{\mu }}_{i}+\frac{d\varphi_{T}}{d\mu }\left(\mu_{i}\right)+2\beta_{R}k_{LT}R_{LT}\left(\lambda_{i},\mu_{i}\right)+\frac{\partial {\text{φ}}_{TA,i-1}}{\partial \mu_{i}}\left(\mu_{i},\mu_{i-1}\right)+\frac{\partial {\text{φ}}_{TA,i}}{\partial \mu_{i}}\left(\mu_{i},\mu_{i+1}\right)=0$$ (36)

are simultaneously solved with the boundary condition, where one end of the myofibril is fixed and the other end is connected to a fixed linear spring (Figure 4B). The parameter $\gamma_{T}$ is the sarcomere friction in the transverse direction, and $\varphi_{T}$ is the deformation energy for stretching in the transverse direction (Washio et al., 2019). In our myofibril model, the rapid sarcomere lengthening in SPOCs is reproduced by the avalanche of reverse strokes at the peak contractile phase in which the population of MHs with large mechanical loads is high. From the SL-LS relationship (Eq. 32) and LS alignment with the adjacent sarcomeres (Eq. 33), the rapid lengthening of one sarcomere transversally compresses the neighboring half-sarcomere in the peak contraction phase. Then, from the SL-LS relationship, the transverse compression enlarges the longitudinal stretch, resulting in the load increments of the attached MHs. As a result, an avalanche of reverse strokes is triggered. In this way, the rapid lengthening wave travels in the myofibril model.

In the finite element ventricle model, the half-sarcomere model is imbedded in each tetrahedral element along the fiber orientation f represented by a unit vector in the reference configuration (Figure 4C). Thus, the half-sarcomere models pull each other at the element interfaces in the fiber orientation. As depicted in Figure 4C, the angle of the fibers relative to the equatorial plane varies depending on the depth of the wall, so that the direction of force developed in the wall covers a wide range. In the finite element analysis, the current position of each material point  X∈Ω in the reference (unloaded) configuration is represented by x=x(X) . Therefore, the stretch in the fiber orientation is given by

λ=∥Ff∥, (37)

where  F=∂x/∂X is the deformation gradient tensor. The active stress $S_{act}$ (as the second Piola-Kirchhoff stress tensor) is formulated based on considering the virtual work done by the active tension $T_{act}$ in the fiber orientation per unit volume in the reference space as follows:

$$\delta w_{act}=-T_{act}\delta \lambda =-\frac{T_{act}}{\lambda }\frac{1}{2}\delta \left(f⊗f:F^{T}F\right)=-\frac{T_{act}}{\lambda }f⊗f:\mathrm{\delta E},$$ (38)

where $\mathrm{\delta E}=\frac{1}{2}\left(\delta F^{T}F+F^{T}\mathrm{\delta F}\right)$ is the infinitesimal increment of the Green–Lagrange strain tensor:

$$E=\frac{1}{2}\left(F^{T}F-I\right).$$ (39)

As a result, with the active stress represented by

$$S_{act}=\frac{T_{act}}{\lambda }f⊗f,$$ (40)

the virtual work by the active stress per unit volume is given by

$$\delta w_{act}=-\mathrm{\delta E}:S_{\mathrm{act}}.$$ (41)

The equation of motion for the displacement, u(X)=x(X)−X , is given as:

∫Ωδu⋅ρu¨ dΩ+∫ΩδE:S dΩ−PL∫ΓLδu⋅n dΓL−PR∫ΓRδu⋅n dΓR=0. (42)

Here, $P_{L}=LVP$ and $P_{R}=RVP$ are the blood pressures in the left and right ventricular cavities, respectively. Ω is the muscle domain in the reference configuration, whereas ${\text{Γ}}_{L}$ and ${\text{Γ}}_{R}$ are the blood–muscle interfaces of the left and right ventricles, respectively, in the configuration at time T , and n is the normal unit vector directed from the cavity to the muscle at these surfaces. The Dirichlet boundary condition u(X)≡0 is imposed on the boundary nodes around the valve rings. The second Piola–Kirchhoff stress tensor S consists of the active, passive, and viscous stresses:

$$S\equiv S_{\text{act}}+S_{\text{pas}}+S_{\text{vis}},$$ (43)

where $S_{\text{act}}$ is given by Eq. 40, and $S_{\text{pas}}=\frac{\partial W_{pas}}{\partial E}$ and $S_{\text{vis}}\;$ are the passive and viscous stresses, respectively. The ventricle blood pressures $P_{L}$ and $P_{R}$ are determined through their interactions with the circulatory systems of the body and the lung. See details of the deformation potential $W_{pas}$ , the viscous stress $S_{\text{vis}}$ , and the coupling with the circulatory system in our previous work (Yoneda et al., 2021).

Assuming a periodical solution over a heartbeat, the time integration on a cycle with the substitutions of $\dot{u}$ and $\dot{E}$ , respectively, into δu and δE in Eq. 42 gives the relationship between the cardiac output $C_{out}$ and the energy production inside the cardiac muscle as follows:

$$C_{out}\equiv -\oint P_{L}{\dot{V}}_{L}dT-\oint P_{R}{\dot{V}}_{R}dT=-\oint \int_{\text{Ω}}\dot{E}:\left(S_{\mathrm{act}}+S_{\mathrm{vis}}\right)\;d\text{Ω}dT.$$ (44)

Here, the inertia and the passive energy terms disappear for the sake of the periodicity assumption. If the viscous energy loss is negligible, the following work done by the active stress is almost equal to the cardiac output.

$$W_{act}\equiv -\oint \int_{\text{Ω}}\dot{\lambda }\;T_{act}d\text{Ω}dT=-\oint \int_{\text{Ω}}\dot{E}:S_{\mathrm{act}}\;d\text{Ω}dT∼C_{out}$$ (45)

**FIGURE 7:**
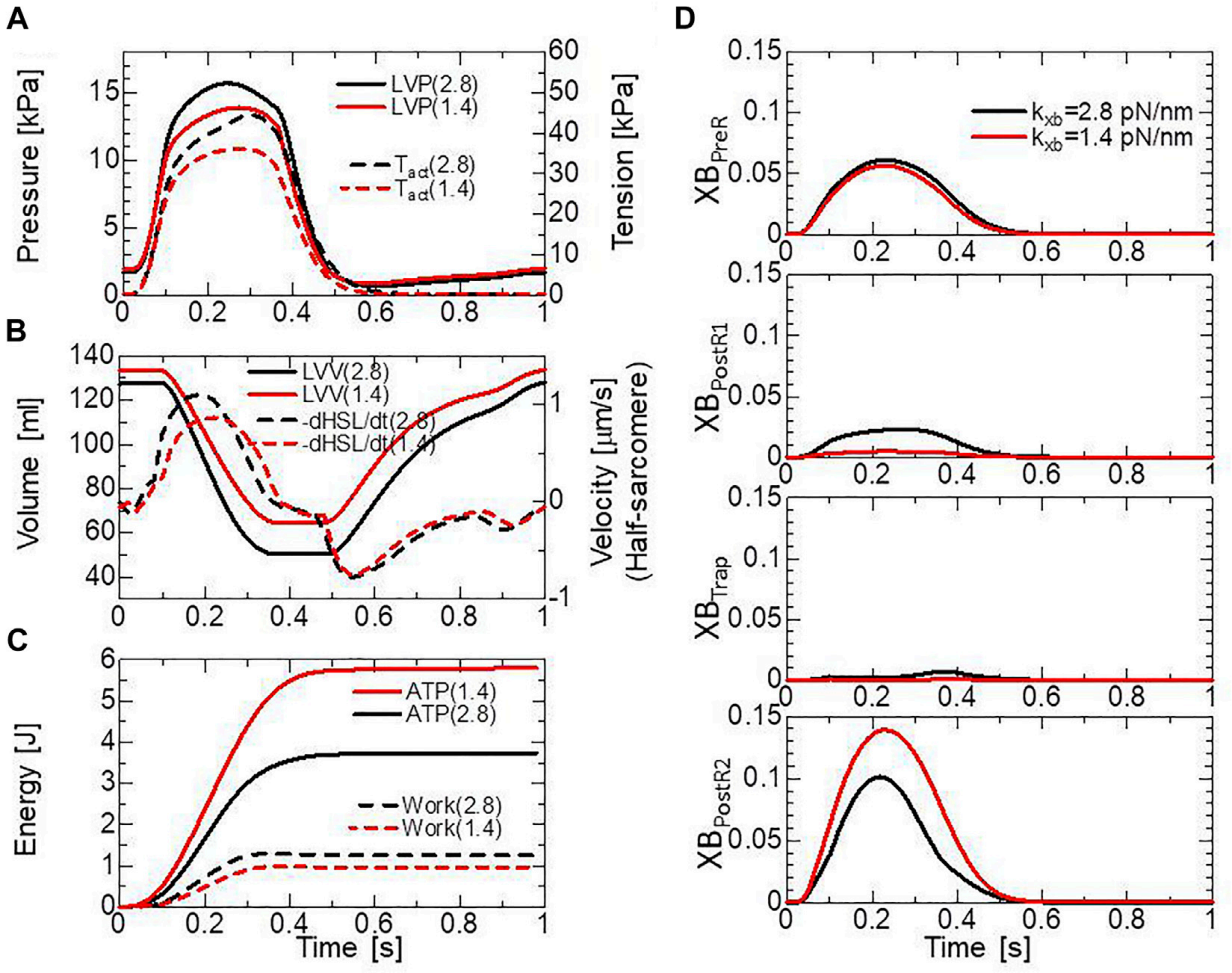
The comparison of pumping heart performance for different myosin rod distortion stiffnesses. The spring constants tested were $k_{xb}$ = 2.8 pN/nm (black lines) and $k_{xb}$ = 1.4 pN/nm (red lines). **(A)** Time courses of the left ventricular pressure (solid lines) and the average active tension (broken lines) over the left ventricular wall (free wall and septum). **(B)** Time courses of the left ventricular volume (solid lines) and the average half-sarcomere shortening velocity over the left ventricular wall. **(C)** Time courses of cumulative energy consumptions (solid lines) and cumulative works (broken lines) by the active tension over the left ventricular wall. **(D)** Time courses of the population ratio of attached MHs in the left ventricular wall classified according to the attached states.

The above relationship between the blood dynamics and muscle dynamics of the left ventricle (the left ventricular free wall and the septum) in our ventricle model is depicted in Figures 7A,B, where the two cases of the myosin rod stiffness with $k_{xb}$ = 2.8 pN/nm (black lines) and $k_{xb}$ = 1.4 pN/nm (red lines) are compared. The solid lines and broken lines represent, respectively, the blood dynamics and the muscle dynamics. Roughly, in the mid-systole ([0.15 s, 0.2 s]), the average active tension reaches 30–40 kPa, with a systolic blood pressure of 12–15 kPa and an average half-sarcomere shortening velocity of 1 μm/s (see also Supplementary Video 2). The relationship between the active tension and the systolic pressure agrees with the simple estimation given by the Laplace law assuming the dimension of the left ventricle in the early-systole (40 mm-diameter, 10 mm-wall thickness, the fiber helix angle twisting from −60° (epicardium) to +60° (endocardium). For example, to support blood pressure of 15 kPa, the active tension $T_{act}$ must be roughly equal to $15\text{ kPa }\times \left(20\;\text{mm}/10\;\text{mm}\right)\times \frac{2\pi }{3\sqrt{3}}\;∼36\;\text{kPa}$ . Here, the factor $\frac{2\pi }{3\sqrt{3}}$ comes from considering the fiber helix angles. The complex distributions of the active tension and the half-sarcomere shortening velocity over the ventricular wall (see Supplementary Video 2) make it difficult to find the correspondence to the tension-shortening velocity relationship under isotonic conditions (Figure 6B). Although the cardiac outputs and the muscle works of the two cases (the solid lines in Figures 7A,B, and the broken lines in Figure 7C) are not so different, the difference in energy consumption (the solid lines in Figure 7C) is remarkable, as indicated also in Figure 6D. The comparison of the time transients of the population ratio of the binding states (Figure 7D) indicates a shorter average dwell time in the XB_PostR1_ state and a larger population in the XB_PostR2_ state for the case of $k_{xb}$ = 1.4 pN/nm than those for the case of $k_{xb}$ = 2.8 pN/nm, resulting in the remarkable energy loss. Therefore, in this study, we analyzed the effects of the trapping mechanism when $k_{xb}$ = 2.8 pN/nm.

## Results

### Stretch activation in the half-sarcomere model

**FIGURE 8:**
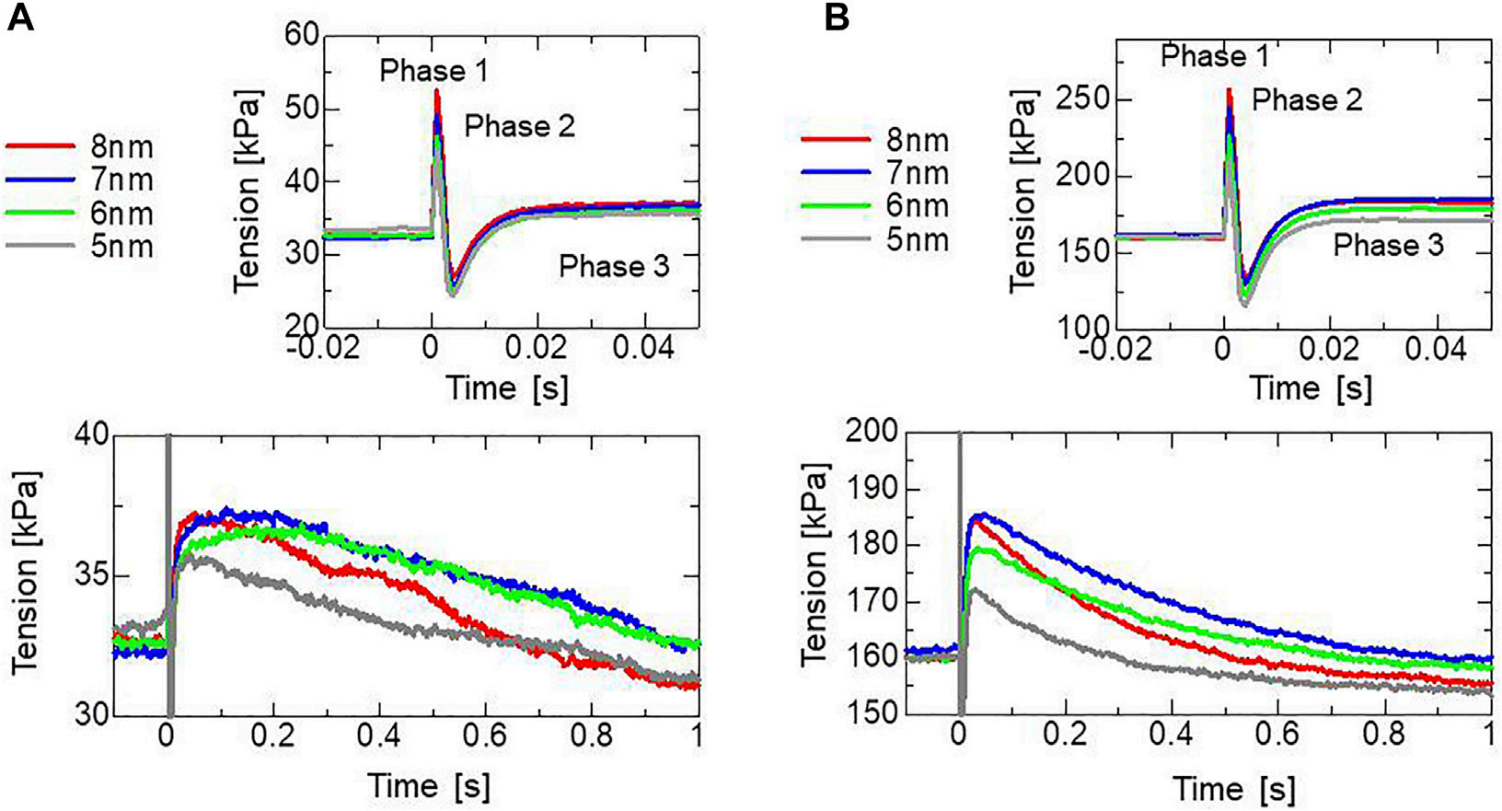
Numerical results of the stretch activation simulation. Stretches of 5, 6, 7, or 8 nm were applied over the time span of 1 ms at T=0 s after the isometric force had matured under constant [Ca^2+^] = 0.3 μm **(A)** and [Ca^2+^] = 0.4 μm **(B)**. Time courses of the tensions are color-coded by the stretching as follows: 5 nm (gray), 6 nm (light green), 7 nm (blue), and 8 nm (red). Enlarged views of the top, focusing on the tension decrease on the order of seconds are depicted on the bottom.

To assess the effectiveness of the trapping mechanism, a stretch-activation test was performed for a single half-sarcomere model consisting of 32,768 filament pairs. Here, stretch lengths of 5, 6, 7, or 8 nm were applied over a 1-ms time interval after the active tension had sufficiently matured. During the simulations, the Ca^2+^ concentration ([Ca^2+^]) was held at a constant value of 0.3 μM (Figure 8A) or 0.4 μM (Figure 8B). A greater increase in tension (the maximum in Phase 3) was observed as the stretching increased up to 7-nm stretch, corresponding to roughly +13% for a 6-nm stretch and +15% for a 7-nm stretch under [Ca^2+^] = 0.3 μm. Here, 7 nm is 0.7% of the half-sarcomere length. These increases lasted for a few seconds after the rapid stretching. In agreement with the experimental results given by Stelzer et al. (2006), our numerical results reproduced “phase 2 (Figure 1A)” in which the force decreased to the steady-state level before stretching. The increase in tension observed by Stelzer et al. (2006) was roughly 16% for a 2% stretch and 8% for a 1% stretch. The tension increases stopped at an 8-nm stretch in the simulation result. In the numerical model, the stretch is directly linked to the shift of the thin filament relative to the thick filament (Figure 3A), while the stretch of the sarcomere may be relaxed by intermediate substances such as Z-lines or intercalated disks in the experimental conditions, as analyzed in the Discussion Section 4.3. Note that we assumed a cross-bridge model under physiological conditions identical to those of a living heart, which differ from the experimental conditions in many aspects, such as temperature.

**FIGURE 9:**
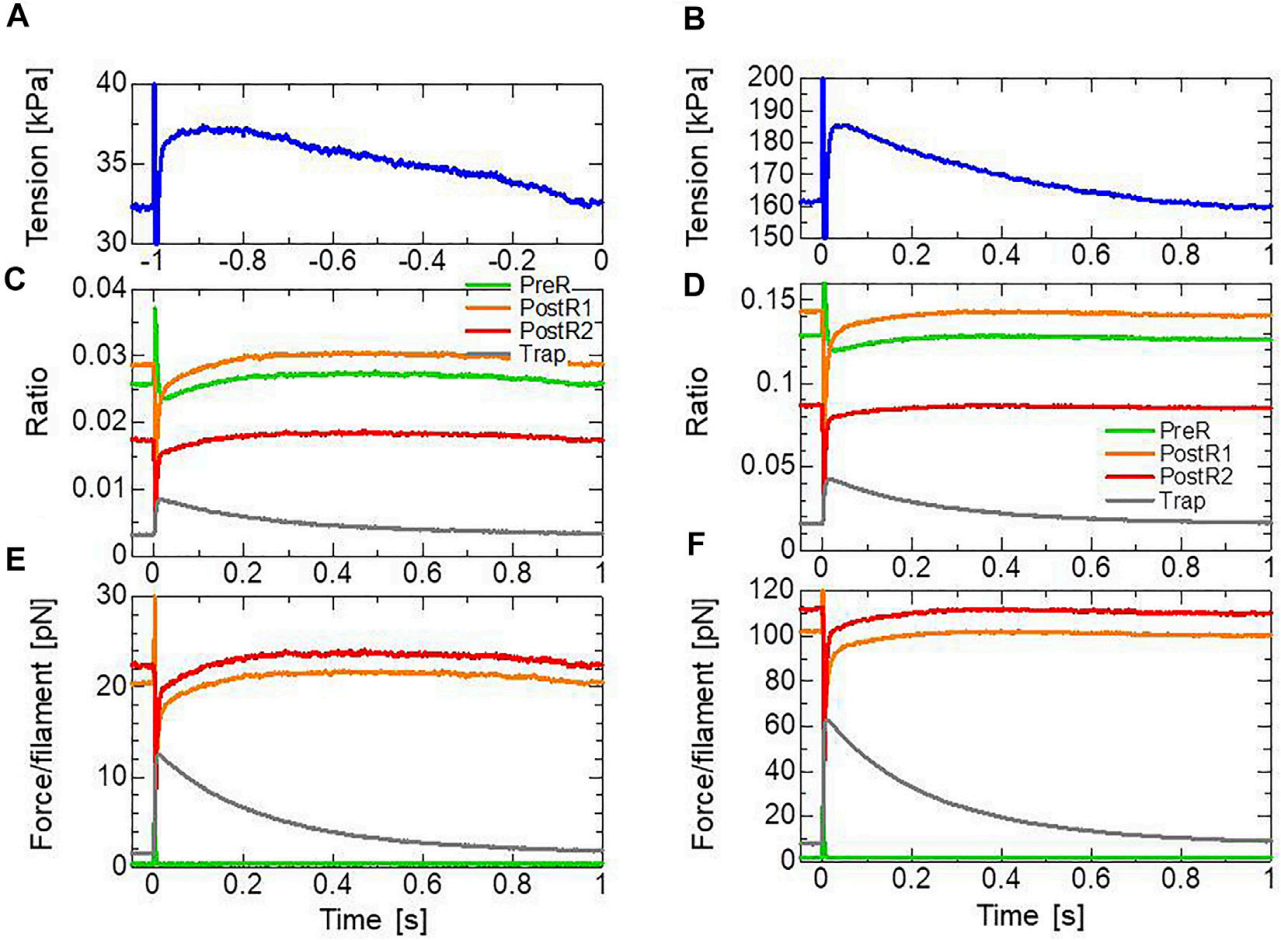
Transients of the four attached states during and after the 1-ms stretching in the stretch activation test with [Ca^2+^] = 0.3 μm (left) and [Ca^2+^] = 0.4 μm (right). **(A,B)** Time courses of the tension for a 7-nm stretch. **(C,D)** Time courses of the population ratio of the attached states: XB_PreR_ (green), XB_PostR1_ (orange), XB_PostR2_ (red), and XB_Trap_ (gray). **(E,F)** Time courses of the force per thin filament in each state.

Focusing on the result for a 7-nm stretch (Figures 9A–F), compared with the pre-stretch steady state, the lasting increase in tension apparently arose from a lasting increase in the population of the XB_Trap_ state (Figures 9C,D). The effect of the XB_Trap_ state is more emphasized when the individual contributions in the active tension of the attached states are plotted (Figures 9E,F). In the half-sarcomere model, we assumed that one thin filament is surrounded by 76 (=  38 ×2 ) myosin molecules. In the steady state before stretching, filament forces of roughly 20 and 22 pN are generated by myosin molecules in the XB_PostR1_ (2.9%) and XB_PostR2_ (1.7%) states, respectively, when [Ca^2+^] = 0.3 μm. Thus, the average force per molecule is 9.1 and 17.0 pN, respectively, in the XB_PostR1_ and XB_PostR2_ states. Because we assumed a force constant of $k_{xb}=$ 2.8 pN/nm for the myosin rod distortion x , the above forces are generated by distortions of 3.3 and 6.1 nm, respectively. After the stretch, a filament force of 12 pN is produced by the myosin molecules in the XB_Trap_ state (∼0.9%). Thus, the average force is roughly 17.5 pN per trapped myosin molecule. This average force is slightly larger than experimentally observed maximal forces (∼15 pN) (Hwang et al., 2021). As analyzed in Section 2.4 and Section 2.5, our setting of the force constant ( $k_{xb}$ = 2.8 pN/nm) seems reasonable with respect to the efficiency in both the isotonic contraction and heart pumping. Furthermore, it may be difficult to measure the maximal force produced by a single myosin molecule under the sarcomeric condition. In particular, the force measured in the filament direction depends on the angle between the myosin rod (S2) and the thick filament, and the maximal force under a similar condition to the stretch activation environment has not been reported.

As observed by Stelzer et al. (2006), the MC cross-bridge model also reproduced the different behaviors in Phase 3 (Figure 1A) in the different activation levels. In our model, this difference is made by the differences in the time courses of the population ratio of the attached states (Figures 9C,D) that are produced by the cooperativity effects, as shown in Figure 5A.

### Effects of the trap mechanism on SPOCs in a single myofibril model

**FIGURE 10:**
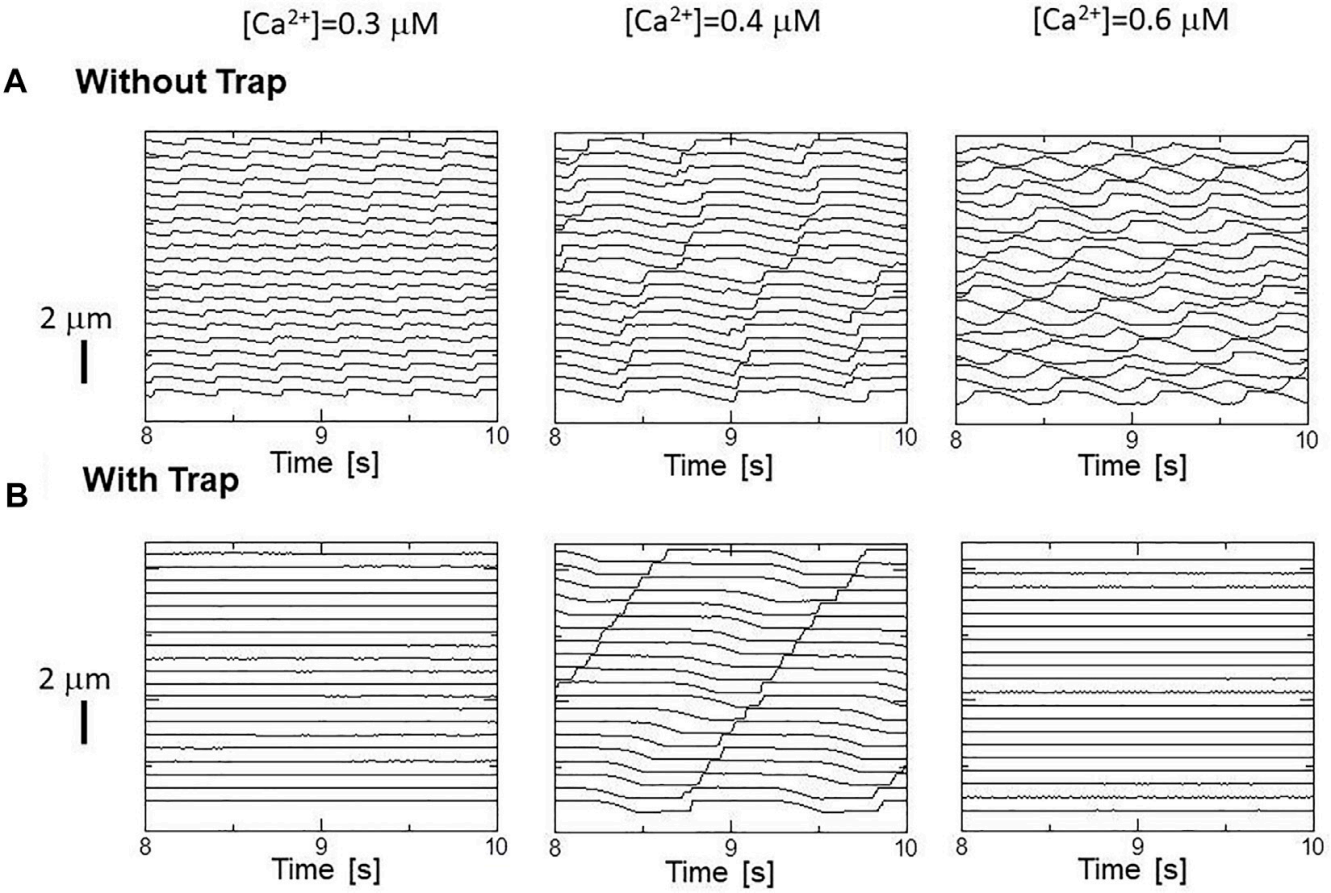
Sarcomere length changes in the myofibril model under activations with different constant calcium concentrations [Ca^2+^] = 0.3 μM  (left), 0.4 μM (center), 0.6 μM (right), for **(A)** the no-trap model and **(B)** the trap model.

**FIGURE 11:**
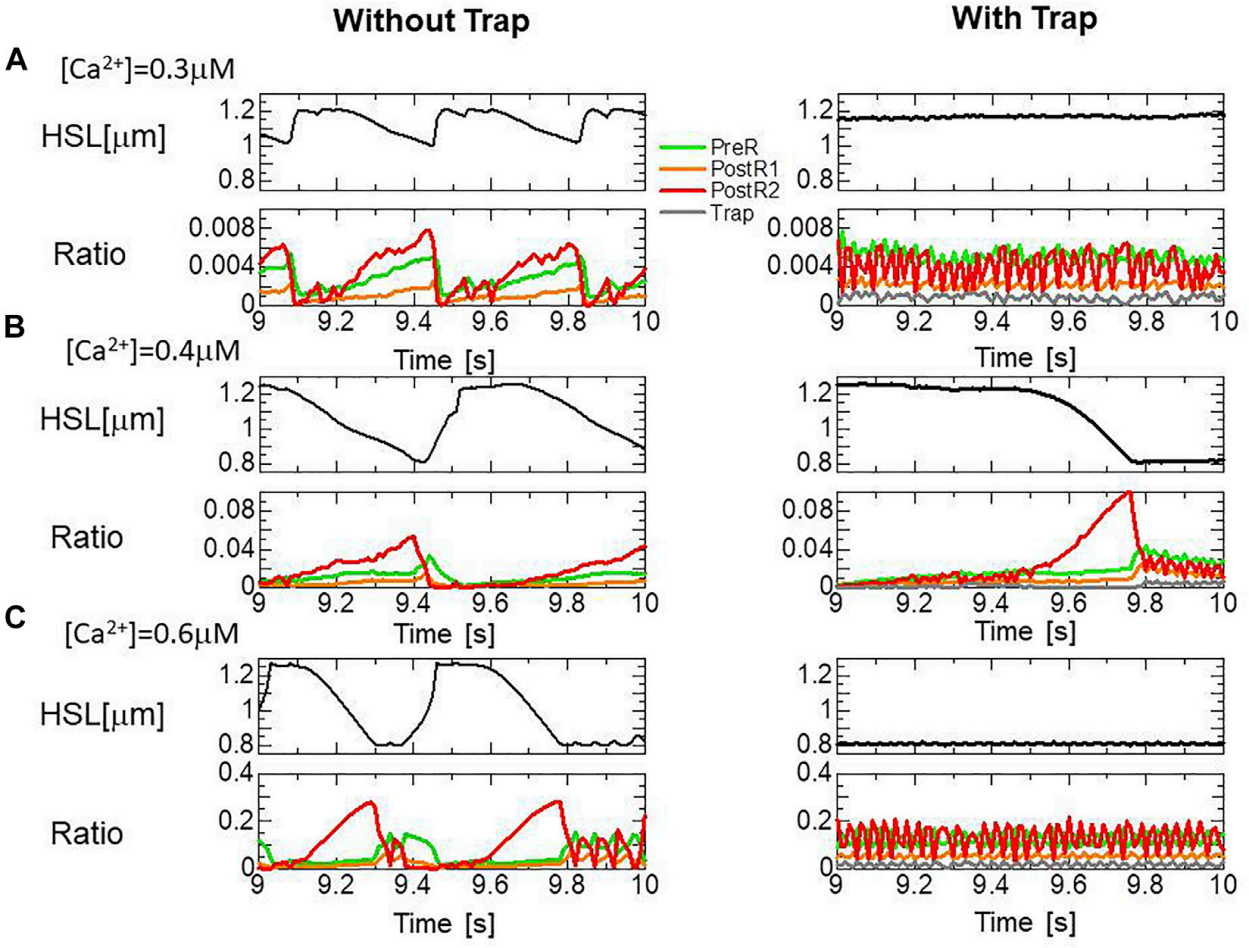
Transitions of the four attached states for the no-trap model (left) and the trap model (right) during SPOCs in a half-sarcomere model imbedded in the myofibril model. Time courses are shown for the half-sarcomere length (black) and the population ratio of binding states: XB_PreR_ (green), XB_PostR1_ (orange), XB_PostR2_ (red), and XB_Trap_ (gray), for the no-trap model (left) and the trap model (right). **(A)** [Ca^2+^] = 0.3 μM **(B)** [Ca^2+^] = 0.4 μM , and **(C)** [Ca^2+^] = 0.6 μM .

The effects of the trap mechanism on SPOCs of a single myofibril model consisting of 40 half-sarcomeres were investigated (Figures 10A,B). Here, 2048 filament pairs were imbedded in each half-sarcomere model. SPOCs were produced for all Ca^2+^ concentrations ([Ca^2+^]) in the no-trap model. In contrast, SPOCs were reproduced only for the intermediate concentration ([Ca^2+^] = 0.4 μM ) between the states of relaxation and contraction in the trap model, as observed by Fabiato and Fabiato (Fabiato and Fabiato, 1978). Therefore, the trap mechanism may contribute to the [Ca^2+^] dependence of the SPOCs by inhibiting the reverse stroke for certain amounts of myosin molecules in the XB_PostR1_ state. For the low Ca^2+^ concentration ([Ca^2+^] = 0.3 μM ), the lengthening was prevented by myosin molecules trapped in the XB_Trap_ state despite the oscillating XB_PostR2_ concentration in the trap model, as indicated by a small increase in the XB_Trap_ state for each reduction in the XB_PostR2_ state (Figure 11A). For the intermediate Ca^2+^ concentration ([Ca^2+^] = 0.4 μM ), the SPOCs are similar for both cases (Figure 11B). For the high Ca^2+^ concentration ([Ca^2+^] = 0.6 μM ), the sarcomeres were shortened to the minimum length, which is almost equal to the thick filament length of *LM* ∼ 1.6 μM , as shown in Figure 1B. At this length, the population of the attached MHs is small because of the small single overlap region of the two filaments. Thus, lengthening was prevented in the same way as that observed for the low Ca^2+^-concentration case in the trap model (Figure 11C).

### Effects in beating ventricle simulation

**FIGURE 12:**
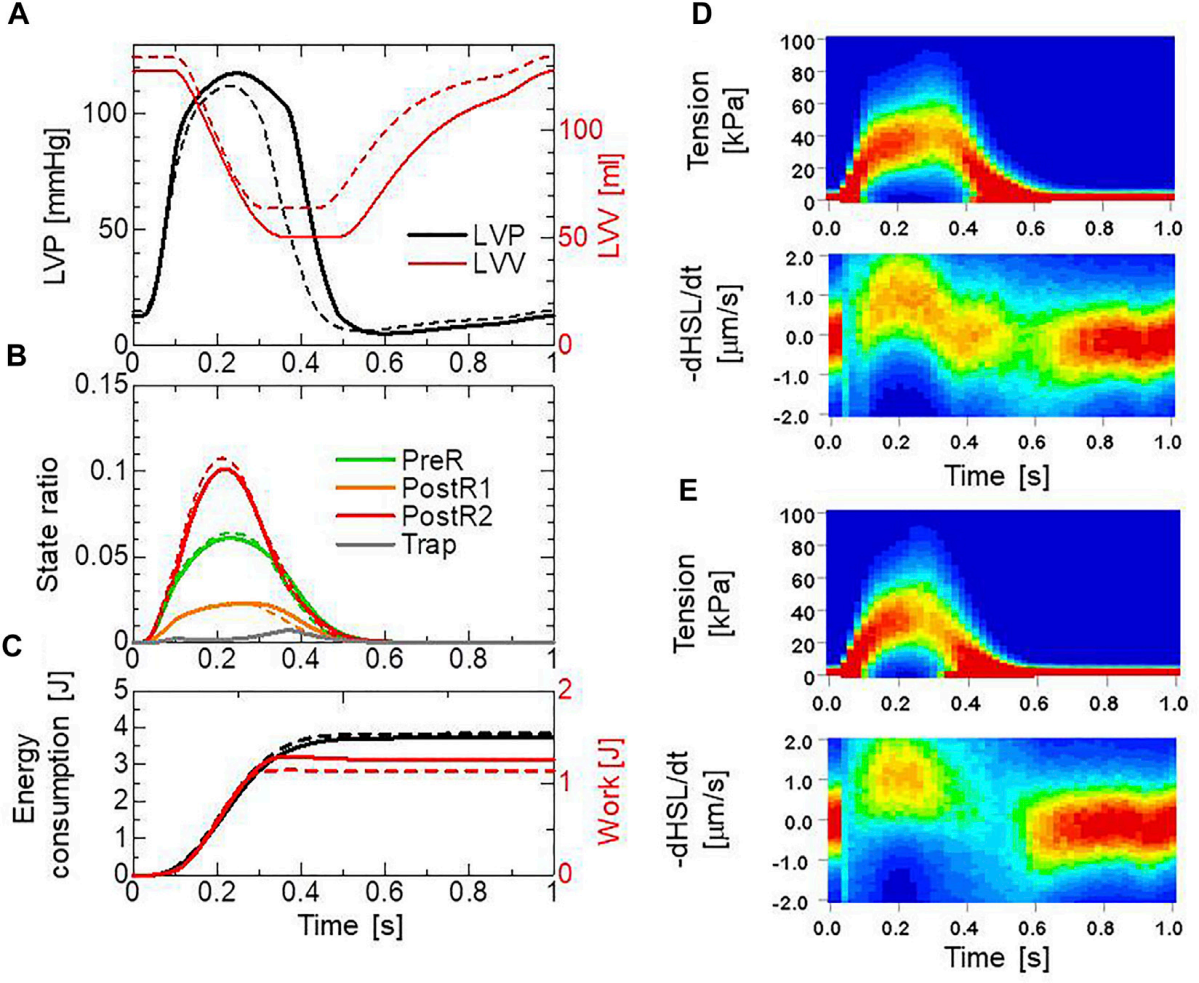
Numerical results of the beating ventricle simulation using the bi-ventricular FEM. **(A)** Time courses of the left ventricular pressure (black) and volume (red) for the no-trap MH model (broken lines) and the trap model (solid lines). **(B)** Time courses of the population ratio of attached MHs in the left ventricular wall classified according to the attached states: XB_PreR_ (green), XB_PostR1_ (orange), XB_PostR2_ (red), and XB_Trap_ (gray), for the no-trap model (broken lines) and the trap model (solid lines). **(C)** Time courses of the cumulative ATP energy consumption (black) and work (red) in the left ventricular wall for the no-trap model (broken line) and the trap model (solid line). **(D)** Contours of the tension distribution (upper) and the half-sarcomere shortening velocity (lower) for the trap MH model. **(E)** Contours of the tension distribution (upper) and the half-sarcomere shortening velocity (lower) for the no-trap MH model.

Beating-ventricle simulations were performed using a finite element ventricle model with the same setup as our previous work (Yoneda et al., 2021). In each element, a sarcomere model consisting of 16 filament pairs was imbedded along the appropriate fiber orientation f . The distribution of the fiber orientations was found by an optimization algorithm based on the isovolumetric active tension (Washio et al., 2020) according to the impulses given by the active tension. Portions of the helical fiber structure are depicted in Figure 4C. The heart rate was set to 60 beats per minute, and the Ca^2+^ transient (Figure 4C) generated by the mid-myocardial cell model proposed by ten Tusscher panflov, (2006) was applied. Transmural delays of the Ca^2+^ transient determined by the distances from the endocardial surfaces of the left and right ventricles under a transmural conduction velocity of 52 cm/s, as measured by Taggart et al. (2000), were adopted. By comparing the trap and no-trap models in Figures 12A,B, one can see that the trap mechanism contributes to maintaining both the high pressure in the last half of the systolic phase and the rapid pressure decrease at the end of the systolic phase (Figure 12A). As a result, the blood volume ejected from the left ventricle in the trap model increased from 71 to 78 mL , while the ATP energy consumption of the left ventricular wall was almost equivalent (Figure 12C). This trend implies that the trap mechanism increases blood ejection without increasing energy consumption. It should be noted that the ATP consumption rates were computed by counting the detachments of MHs in the XB_PostR2_ state transferred to those in the N_XB_ state, which was controlled by the rate constant $r_{NXB}$ and the forced detachments defined by Eqs 14 and 15. As shown in Figures 12A,B slight increase in the population of MHs in the XB_Trap_ state can be seen at the end of the systolic phase (Figures 12D,E). This increase corresponds to a prolonged ejection time in the trap model, as shown in Figure 12A.

**FIGURE 13:**
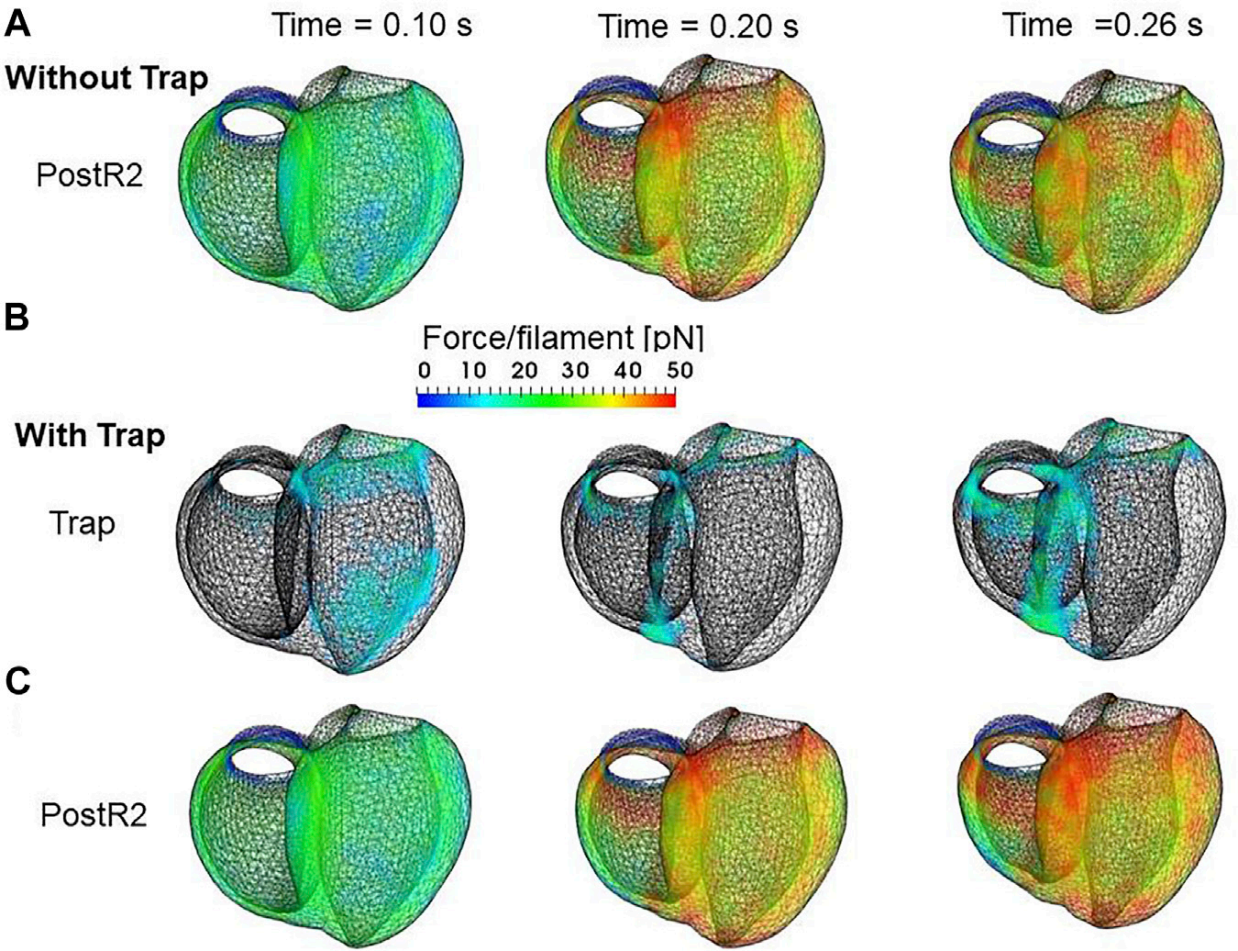
Distribution of contraction forces in the ventricles in the systolic phase at the early systole: 0.10 s (left), at the mid-systole: 0.20 s (center), and at the end-systole: 0.26 s (right) for **(A)** the XB_PostR2_ state in the no-trap model **(B)** XB_Trap_, and **(C)** the XB_PostR2_ state in the trap model. In **(B)**, the regions in which the forces are less than 10 pN are transparent.

**FIGURE 14:**
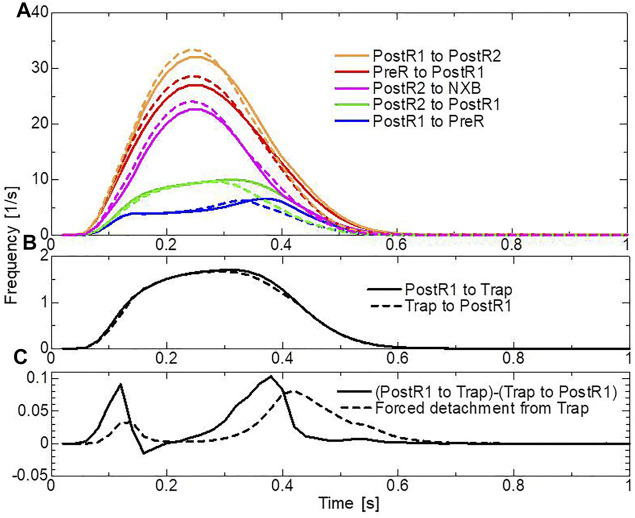
Transition frequencies of binding MHs during a heartbeat. **(A)** The frequencies per MH of the transitions from XB_PostR1_ to XB_PostR2_ (orange), XB_Pre_ to XB_PostR1_ (red), XB_PostR2_ to N_XB_ (pink), XB_PostR2_ to XB_PostR1_ (green), and XB_PostR1_ to XB_PreR_ (blue). The solid and broken lines represent the trap model and the no-trap model, respectively. **(B)** Frequencies per MH of trapping (solid line) and escaping (broken line). **(C)** The difference between the trapping frequency and the escaping frequency (solid line), and the frequency of the forced detachment from XB_Trap_.

In the systolic phase, the cardiac myocytes within the shared fiber bundle support each other by pulling their neighbors *via* contractile forces, with the active tension in Eq. 22 playing the greatest role. Therefore, based on the mechanical equilibrium condition in the fiber direction, the active tensions must be almost equal. Consequently, if there is a loss of active tension at one point of the fiber bundle prior to the remaining parts in the early-systole or in the end-systolic phase, this portion would be lengthened quickly, and the sarcomeres in the remaining parts would be shortened until a mechanical equilibrium is reached. Because this transition accompanies decreases in the active tension owing to the loss of distortion in the myosin rods, stopping this process as early as possible is desirable to maintain blood pressure. The trapping mechanism can achieve this goal, as shown in Figures 13A–C, which depict the distributions of the active tension generated by the individual states XB_PostR2_ and XB_Trap_ in the end-systolic phase. The degenerated forces of the XB_PostR2_ state in the no-trap model (Figure 13A) are compensated by the force generated by the XB_Trap_ state in the trap model (Figure 13B). Furthermore, the forces of the XB_PostR2_ state in the trap model around the degenerated regions in the no-trap model (Figure 13C) are well maintained, owing to the contributions of the reinforced regions *via* the trap mechanism. As shown in the frequencies of transitions (Figure 14), the reverse power stroke contributes to diastolic relaxation to the same extent as the detachment from the XB_PostR2_ state (Figure 14A). Although the difference between the trapping and escaping frequencies averaged over the left ventricular wall (Figure 14B) is relatively small, the two peaks of the difference in Figure 14C agree with the differences in LVP between the trap model and the no-trap model in Figure 12A. Figure 14C also indicates that a certain extent of the trapped MHs is forced to detach because of the extreme distortion (Eq. 15).

## Discussion

### Effects of the trapping mechanism

In the stretch activation tests, the step length dependence was reproduced (Figure 8), similar to the experimental results of Stelzer (Stelzer et al., 2006). However, the minimum values for the rapid force decay (phase 2) in the numerical results were lower than the original isometric force before stretching and higher than the isometric force in the experimental results of Stelzer. Conversely, minimum values smaller than the original isometric force were reported for wild-type myocardium by Mamidi et al. (Mamidi et al., 2018). Thus, the level of the minimum force in phase 2 compared with the original isometric force seems to depend on the experimental conditions. In our model, the decay was determined from decreases in the XB_PostR1_ and XB_PostR2_ states owing to the reverse strokes from XB_PostR2_ to XB_PostR1_ and XB_PostR1_ to XB_PreR_. Meanwhile, the loss of force was compensated by the increase in population and the distortions of the trapped myosin molecules in the XB_Trap_ state (Figures 9B,C). Here, the rates of reverse strokes are limited by the upper bounds ${\bar{r}}_{b,i}$ , as in Eq. 19, and the population in the XB_Trap_ state is determined by the parameter values associated with the trap mechanism ( $s_{trap}$ , $h_{\text{trap}}$ , and $h_{\text{escape}}$ ) in Eqs 3 and 4 and the forced detachment ( $x_{XB,t}$ , $c_{XB,t}$ , and $a_{XB,t}$ ) in Eq. 15. In our trap model, these parameters were carefully chosen, focusing not only on the stretch activation phenomena, but also on the SPOCs and performance in the beating ventricle model.

In our model, the trap mechanism was added to the XB_PostR1_ state, which is the state after the first power stroke. Alternatively, it might be conceivable to add a similar trap mechanism in the XB_PostR2_ state (the state after the second power stroke) if a force-dependent detachment rate constant (Greenberg et al., 2014) is adopted in the numerical model. Hwang et al. (2021) reported a much gentler increase in the reverse stroke rate constant of the first stroke compared with that of the second stroke, with a force increase of 8–14 pN. Their single-molecule experimental results support the adequacy of adding the trap mechanism to the XB_PostR1_ state.

The numerical SPOC results indicate that the trap mechanism affects the calcium activation sensitivity for the relaxation dynamics in an advantageous manner. The trap mechanism prevents sarcomere lengthening at high or low levels of calcium activation (Figure 10). The sarcomere lengthening maximizes the single overlap region between the two filaments, resulting in a facilitation of re-activation. Thus, the prevention of lengthening at low calcium levels (Figure 11A) stops weak re-activation, leading to a smooth relaxation of muscle in the diastolic phase (Figure 12A). Conversely, the prevention of lengthening at high calcium levels (Figure 11C) stops sarcomere shortening in the neighboring cardiomyocytes, causing a retention of high active tension in the fiber bundle (Figures 13B,C).

### Advantages of MC simulations

The above-mentioned features for heartbeats are similar to those found in our previous work (Washio et al., 2018) with the Langevin dynamics model. However, the new MC model produced better results in the tension response for the step length changes and SPOCs owing to the simpler adjustment of related parameters. In a previous study, MC simulations were performed for a three-dimensional half-sarcomere model consisting of three myosin filaments and 13 thin filaments implemented with 360 myosin molecules and 1,170 binding sites to examine the impact of filament compliance on Ca^2+^ activation (Chase et al., 2004). However, these simulations were conducted only under steady-state conditions and were not coupled with a macroscopic finite element model (FEM). Moreover, in the beating ventricle simulation, the computation cost per beat was 1.5 h when 320 cores of a conventional parallel computer system were used for the MC model (Yoneda et al., 2021), while the cost was 105 h when 1920 cores of the same computer system were used (Washio et al., 2018). The former simulation was performed for a bi-ventricular FEM consisting of 45,000 tetrahedral elements with 16 filament pairs, while the latter was performed for a smaller FEM consisting of 7,900 elements with eight filament pairs. The improvements of the MC model, such as those demonstrated in the current study, will lead to better predictions in clinical applications (Kariya et al., 2020).

### Limitations

Smith et al. argued that multiple working strokes are required for a cross-bridge cycle to satisfy energetic constraints and demonstrated that a cross-bridge cycling model with three strokes can reproduce various experimental findings observed for frog muscle, including absolute values of active tension, stiffness, and ATPase rate; the phase-2 tension response to a length release; and the transient tension rise during ramp stretching (Smith et al., 2008a; Smith et al., 2008b). This line of reasoning led us to adopt a model with two strokes with lengths of 6.0 and 4.0 nm to realize the 10-nm stroke suggested by the crystal structure of myosin molecules. In the three-stroke model of Smith et al., the first two strokes, which each have a length of 5.0 nm, occur around the Pi release, and a third small stroke was implemented to account for the strain-dependent ADP release rate. Although we did not explicitly define the relationship between strokes and the nucleotide released in our model, these points should be examined by comparing our results against various experimental findings in future work.

The average force of the trapped MH was larger than the maximal force ever observed experimentally. In our model, this discrepancy comes from our stiffness parameter $k_{xb}$ = 2.8 pN/nm for nearly a 10 nm stretch, assuming linear elasticity. The force function, $F_{rod}$ , in Eq. 20 may be improved by reducing the stiffness for substantial distortions. Further investigation and modification of the detachment rate function in Eq. 15 represent future tasks.

In our model, we didn’t account for the elasticity of the components, such as the thin and thick filaments and the Z-band in the sarcomere. Namely, we assumed that all of the sarcomere components (except for the myosin rods) are rigid. Thus, the macroscopic stretch change Δλ is directly reflected in the distortion increase $\Delta x=SL_{0}\Delta \text{λ}/2$ . However, in the actual setting, the distortion increase might be somewhat relaxed because of the elasticities. For example, if we assume a linear elasticity of the Z-line with a spring constant of $k_{Z}$ and $N_{xb}$ attached myosin molecules per thin filament, we have:

$$\{\begin{array}{c} N_{xb}k_{xb}\Delta x=k_{Z}\Delta Z,\\ \Delta x+\Delta Z=\frac{SL_{0}\Delta \text{λ}}{2}. \end{array}$$ (46)

By eliminating the Z-line distortion increment ΔZ in Eq. 46, we obtain:

$$\left(1+\frac{N_{xb}k_{xb}}{k_{Z}}\right)\Delta x=\frac{SL_{0}\Delta \text{λ}}{2}.$$ (47)

Thus, the distortion increment Δx decreases as the number of attached myosin molecules increases. Here, the magnitude of the distortion increment Δx corresponds to the strength of trapping for myosin molecules in the XB_Trap_ state. Because $N_{xb}$ is smaller for a lower activation, Eq. 47 may give a straightforward explanation of the experimental findings reported by Stelzer (Stelzer et al., 2006), who reported that the stretch activation is most pronounced at low levels of Ca^2+^ activation. Apart from these considerations, the effect of filament compliance on the realignment of cross-bridges reported by Chase et al. (Chase et al., 2004) is an important issue that should be included in future modeling studies.

Razumova et al. modeled and compared three possible mechanisms of cooperativity: 1) interactions between adjacent T/T RUs, 2) interactions between adjacent cycling cross-bridges, and 3) a facilitation of the transition to the on-state of a RU by the adjacent attached cross-bridge, which all control the open and closed states of the thin filament (Razumova et al., 2000). Their results clearly demonstrated distinct roles of these interactions in the maximal force and cooperativity; however, their formulations are conceptual and thus do not represent specific molecular interactions. McKillop and Geeves proposed a three-state (blocked, closed, and open) model of thin filament activation, which is compatible with X-ray diffraction data (McKillop and Geeves, 1993). Smith et al. further attempted to establish the relation between RU–RU interactions and physical entities (Smith et al., 2003) by modeling the flexible-chain-like structure of the tropomyosin molecule with a continuous-flexible-chain model. In this regard, the RU–RU model used in this study is also empirical and lacks a relation to a physical entity. Moreover, only part of the above-mentioned mechanism of cooperativity was considered.

In the finite element ventricular model, a single half-sarcomere model was imbedded in each tetrahedral element, and the half-sarcomere length changed according to the stretch of the element in the fiber direction. This is nothing but assuming that the movements of sarcomeres contained in each element are perfectly synchronized. In reality, there may be time lags in the length changes between the neighboring sarcomeres particularly in the relaxation phase. In our future work, the issue will be studied by using the homogenization method (Washio et al., 2013) whereby the bundle of myofibril models is imbedded in each element.

## Data Availability

The raw data supporting the conclusion of this article will be made available by the authors, without undue reservation.
