# Supplementary material for: A thermodynamically consistent monte carlo cross-bridge model with a trapping mechanism reveals the role of stretch activation in heart pumping
Source: Front Physiol. 2022 Sep 8;13:855303. doi: 10.3389/fphys.2022.855303 (PMC9498833; doi:10.3389/fphys.2022.855303)
Supplement: Supplementary file 1 [file DataSheet1.PDF]

Supplementary Material

A Thermodynamically Consistent Monte Carlo Cross-Bridge Model with a Trapping Mechanism Reveals the Role of Stretch Activation in Heart Pumping

Kazunori Yoneda[1], Ryo Kanada[2] Jun-ichi Okada[3,4], Masahiro Watanabe[1], Seiryo Sugiura[3], Toshiaki Hisada[3], Takumi Washio[3,4*]

[1] Section Solutions Division, Healthcare Solutions Development Unit, Fujitsu Japan Limited, Shiodome City Center, 1-5-2 Higashi-Shimbashi, Minato-ku, Tokyo 105-7123, Japan.

[2] RIKEN Center for Computational Science HPC- and AI-driven Drug Development Platform Division, AI-driven Drug Discovery Collaborative Unit, Kobe 654-0047, Japan.

[3] UT-Heart Inc., Kashiwanoha Campus Satellite, 178-4-4 Wakashiba, Kashiwa, Chiba 277-0871, Japan.

[4] Graduate school for frontier sciences, University of Tokyo, Kashiwanoha Campus Satellite, 178-4-4 Wakashiba, Kashiwa, Chiba 277-0871, Japan.

*** Correspondence:**
Takumi Washio
washio@ut-heart.com

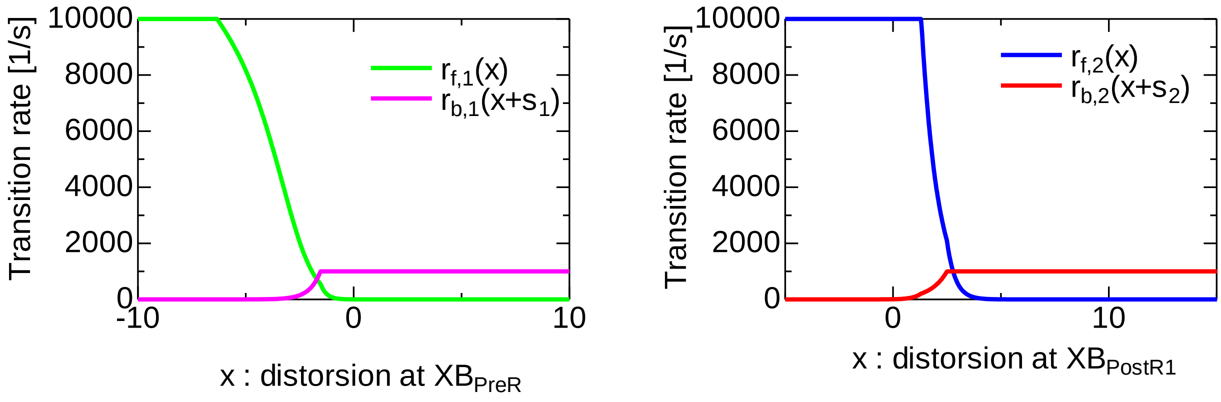

Figure S1 Rate Constants of the power strokes and the reverse strokes

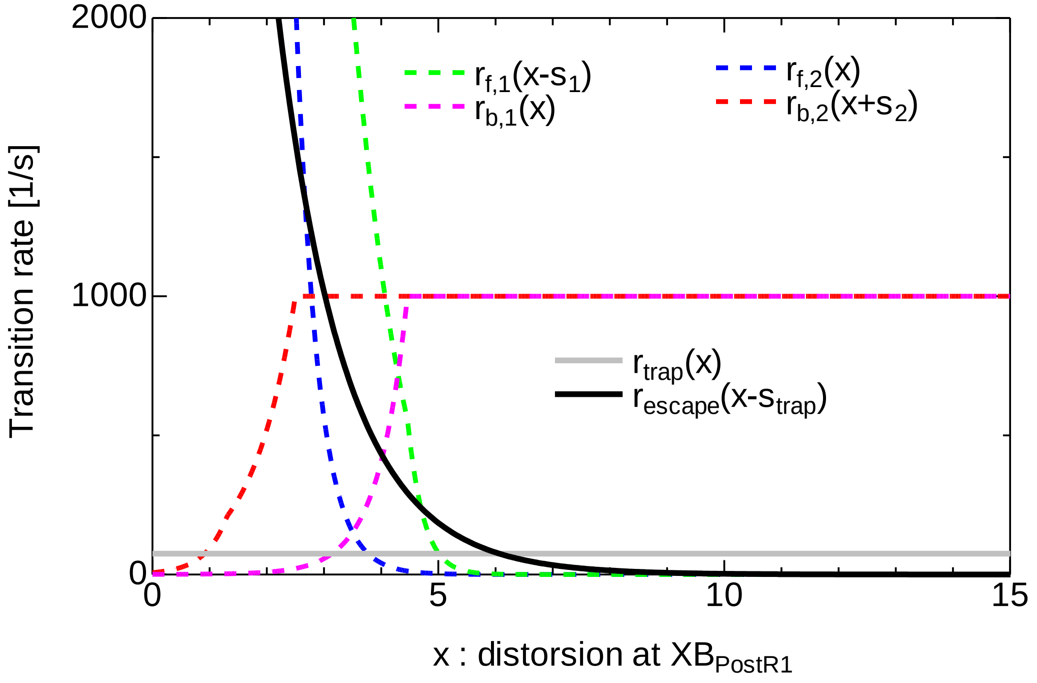

Figure S2 Rate Constants of the trapping and the escaping as the functions of the distortion at XB_{PostR1}
